# mEAK-7 Forms an Alternative mTOR Complex with DNA-PKcs in Human Cancer

**DOI:** 10.1016/j.isci.2019.06.029

**Published:** 2019-06-25

**Authors:** Joe Truong Nguyen, Fatima Sarah Haidar, Alexandra Lucienne Fox, Connor Ray, Daniela Baccelli Mendonça, Jin Koo Kim, Paul H. Krebsbach

**Affiliations:** 1 Department of Biologic and Materials Sciences, University of Michigan, Ann Arbor, MI 48109, USA; 2 Biointerfaces Institute, University of Michigan, Ann Arbor, MI 48105, USA; 3 Section of Periodontics, School of Dentistry, University of California, Los Angeles, Los Angeles, CA 90095, USA

**Keywords:** Biological Sciences, Cell Biology, Cancer

## Abstract

MTOR associated protein, eak-7 homolog (mEAK-7), activates mechanistic target of rapamycin (mTOR) signaling in human cells through an alternative mTOR complex to regulate S6K2 and 4E-BP1. However, the role of mEAK-7 in human cancer has not yet been identified. We demonstrate that mEAK-7 and mTOR signaling are strongly elevated in tumor and metastatic lymph nodes of patients with non-small-cell lung carcinoma compared with those of patients with normal lung or lymph tissue. Cancer stem cells, CD44+/CD90+ cells, yield elevated mEAK-7 and activated mTOR signaling. mEAK-7 is required for clonogenic potential and spheroid formation. mEAK-7 associates with DNA-dependent protein kinase catalytic subunit isoform 1 (DNA-PKcs), and this interaction is increased in response to X-ray irradiation to regulate S6K2 signaling. DNA-PKcs pharmacologic inhibition or genetic knockout reduced S6K2, mEAK-7, and mTOR binding with DNA-PKcs, resulting in loss of S6K2 activity and mTOR signaling. Therefore, mEAK-7 forms an alternative mTOR complex with DNA-PKcs to regulate S6K2 in human cancer cells.

## Highlights

• mEAK-7 forms an alternative mTOR complex with DNA-PK in human cancer
• mEAK-7 is upregulated in the metastasized lymph nodes of patients with NSCLC
• DNA-PK is essential for mEAK-7-mTOR-S6K2 signaling in human cancer
• mEAK-7 is required for cancer cell clonogenicity and spheroid formation

## Graphical Abstract

**Figure:**
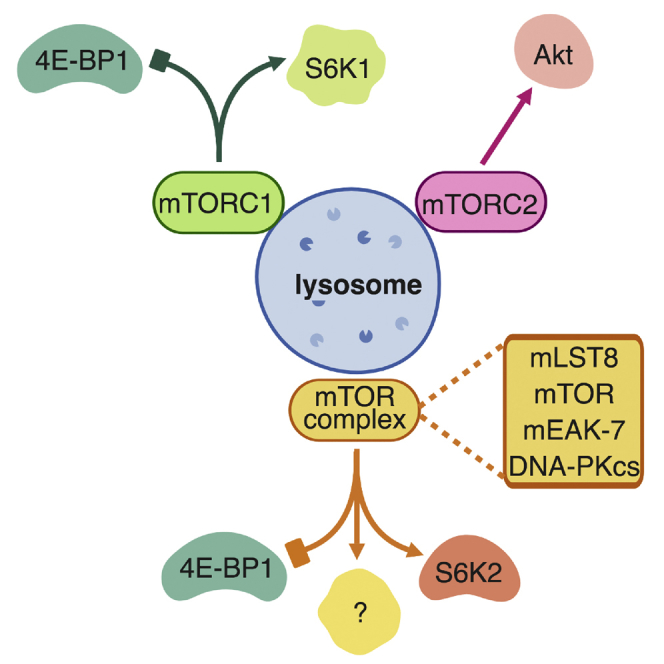

## Introduction

Aberrant mechanistic target of rapamycin (mTOR) signaling has been observed in many types of human cancer (Saxton and Sabatini, 2017). Recently, mEAK-7 (mammalian EAK-7 or MTOR associated protein, eak-7 homolog) was identified as a molecular activator of mTOR signaling in human cells (Nguyen et al., 2018). Interestingly, mEAK-7 exhibits a preferential expression pattern in human cancer cell lines (Nguyen et al., 2018). Although EAK-7 regulates dauer formation and lifespan in *C. elegans* (Alam et al., 2010), the extent to which EAK-7 functions similarly in nematodes and mammals to regulate TOR/mTOR function is unknown.

mEAK-7 uses the S6K2/4E-BP1 axis to regulate mTOR signaling (Nguyen et al., 2018). S6K2 signaling has not been adequately delineated from that of S6K1 signaling owing to their assumed functional redundancies (Pardo and Seckl, 2013). However, in breast cancer cells, loss-of-function studies demonstrate that S6K1 and S6K2 have several different protein targets (Karlsson et al., 2015). In addition, canonical models of mTOR complex 1 (mTORC1), the traditional S6K regulators, and mTORC2 may not exist similarly in all cell types. As examples of this phenomena, an mTOR complex that involves GIT1, which is distinct from mTORC1 and mTORC2, has been identified in astrocytes (Smithson and Gutmann, 2016), and ETS Variant 7 is capable of binding to mTOR and sustaining mTOR signaling in the presence of rapamycin (Harwood et al., 2018). These pivotal findings disrupt conventional ideas regarding the existence of only two mTOR complexes and therefore suggest the possibility of other, unidentified mTOR complexes.

Although it is largely believed that mTOR signaling is suppressed under genotoxic stress via AMPK regulation of TSC2 (Feng et al., 2007), studies have demonstrated aberrant activation of mTOR signaling in response to DNA damage. For example, mTORC1 signaling inhibits DNA damage response mechanisms *in vitro* and *in vivo* through RNF168 (Xie et al., 2018). S6K2, another crucial mTOR target, may also function in the DNA damage response, as S6K2 knockdown results in strong reduction of mTOR signaling, even in the presence of DNA damage (Xie et al., 2018). Furthermore, CHK1 function relies on mTORC1 signaling in response to DNA damage repair processes. These findings suggest that mTOR signaling supports DNA damage responses (Zhou et al., 2017). In examining the role of radiation in DNA damage, sustained radiation treatment to mice activates mTOR signaling and oxidative stress in the intestine (Datta et al., 2014), whereas normal tissues undergoing long-term radiation stress exhibit activated mTOR signaling in mini pigs (Zhu et al., 2016). Thus, there is a rationale to treat patients with a combination of chemotherapeutics that induce DNA damage and mTOR inhibitors, like rapamycin, due to additive cytotoxic effects in breast carcinoma cell lines (Mondesire et al., 2004). These studies suggest that mTOR signaling and DNA damage repair processes may function synergistically in specific biologic contexts, such as during the downregulation of p53 via S6K-mediated activation of MDM2 (Lai et al., 2010), or the phosphorylation of 4E-BP1 phosphorylation in response to DNA damage (Braunstein et al., 2009). Thus, we posit a mechanism supporting sustained mTOR signaling after genotoxic stress, which may allow enhanced cancer cell survival through radiation resistance.

Cancer stem cells (CSCs) are known to be radiation resistant and thrive under genotoxic stress, but the molecular mechanisms responsible for these adaptations remain unknown (Bao et al., 2006, Diehn et al., 2009). CSCs are a self-renewing population of cells within a tumor mass (Al-Hajj et al., 2003), and mTOR signaling has been implicated in regulating pancreatic CSC viability and self-renewal (Matsubara et al., 2013). This suggests that this population of cancer cells utilizes mTOR signaling to contribute to the survival and pathogenicity of human cancers. Data from a medulloblastoma *in vivo* model of CSCs suggest that phosphatidylinositol 3-kinase (PI3K) signaling is activated in response to DNA damage, as indicated by S6 regulation, a crucial readout of mTOR signaling (Hambardzumyan et al., 2008). This substantive evidence suggests that mTOR signaling plays an important role in CSC DNA damage response and self-renewal.

Given that genotoxic stressors are capable of activating mTOR signaling, select CSCs were found to demonstrate radiation resistance, and because CSCs require mTOR signaling, we sought to determine the extent to which mEAK-7 contributes to radiation resistance and self-renewal in cancer cells through an alternative pathway involving mTOR.

## Results

### mEAK-7 Protein Levels Are Elevated in Metastatic Human Non-Small Cell Lung Carcinoma Lymph Nodes

**Figure 1:**
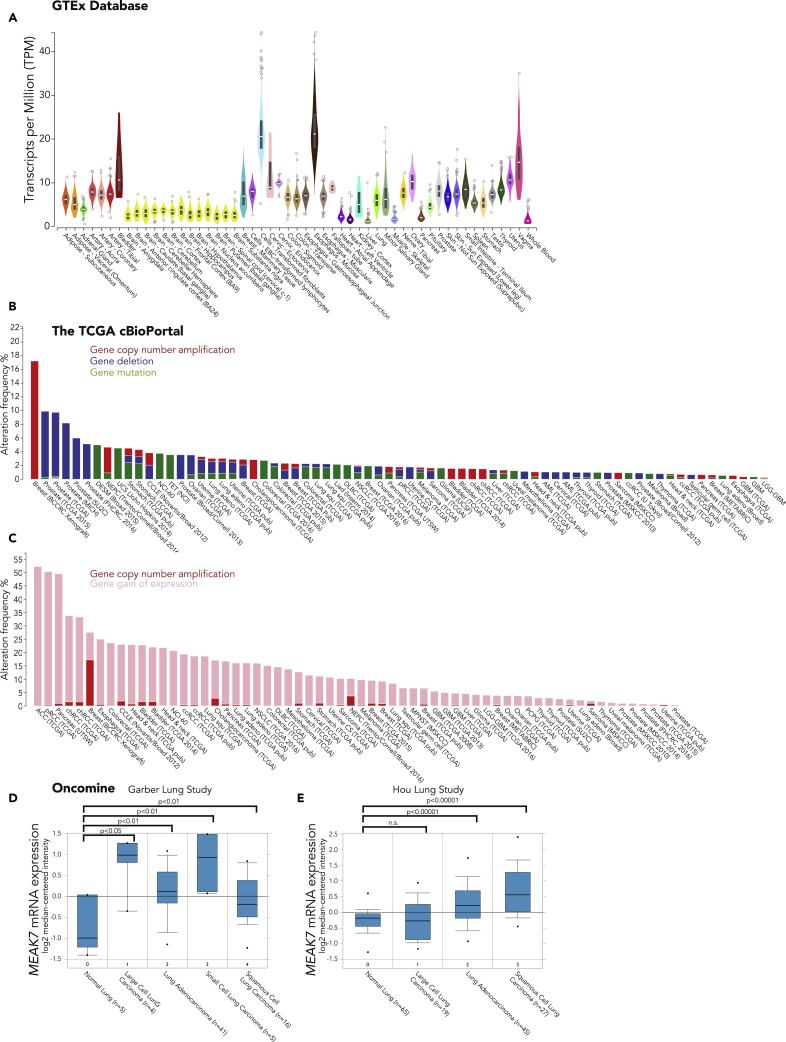
*MEAK7* Gene Expression Is Detected in Normal Human Cells and Upregulated in Select Human Cancer Types (A) Genotype-Tissue Expression (GTEx) database analysis of *MEAK7* expression in normal human tissues. (B and C) (B) The Cancer Genome Atlas cBioPortal analysis *MEAK7* for all genomic alterations or (C) for only gain of mRNA expression or amplification of gene copy number. The results shown are based on data generated by TCGA Research Network: http://cancergenome.nih.gov/. (D and E) Oncomine analysis of *MEAK7* gene expression of patients with normal lung and lung cancer via two different studies: (D) Garber Lung Study analysis by Student's t test 2-sample equal variance and (E) Hou Lung Study analysis by Mann-Whitney U test, p < 0.01, 2-tailed.

Although mEAK-7 protein levels appear to be disproportionately high in human cancer cell lines when compared with non-cancerous cells (Nguyen et al., 2018), this limited observation does not exclude the possibility that mEAK-7 is present in healthy human tissues, because mTOR expression is found in many tissue types (Kim et al., 2002). To gain a better understanding of the expression pattern of mEAK-7 in healthy human tissues, we accessed the GTEx database and identified basal-level expression of *MEAK7* in many human tissues (Figure 1A). The BioGPS database also confirmed that *MEAK7* is expressed in diverse tissue types (Figure S1A) (Wu et al., 2009, Wu et al., 2013, Wu et al., 2016). Thus, future analyses of mEAK-7 in healthy tissues are essential to understanding the role of mEAK-7 in mammalian development and metabolism.

To identify *MEAK7* genomic alterations in human patients with cancer, we accessed the cBioPortal database. Genetic modifications to *MEAK7* included deletions, copy number amplifications, and mutations; yet these modifications were found to be cancer type dependent (Figures 1B and 1C). For example, many prostate cancers exhibit *MEAK7* deletions, whereas breast cancers often sustain substantial *MEAK7* gene copy number amplification (Figure 1B). Because of these different genetic profiles, the search parameters were narrowed to include only gain of expression and copy number amplification. The percentage of patients that demonstrated either of these anomalies ranged between 5% and 50% (Figure 1C) (Cerami et al., 2012, Gao et al., 2013), demonstrating that *MEAK7* genetic modifications are cancer type specific and can be observed in a diverse array of human cancers.

The TCGA cBioPortal revealed high expression of *MEAK7* in patients with non-small-cell lung carcinoma (NSCLC) (Figure 1C). In addition, the majority of our first report into the mechanistic function of mEAK-7 was done in NSCLC lines, H1299 and H1975 (Nguyen et al., 2018). Therefore, the Oncomine database was accessed to analyze *MEAK7* expression patterns in lung carcinomas when compared with expression patterns in healthy lung tissue. Through two different lung cancer studies, *MEAK7* was found to be highly expressed in many NSCLCs and small cell lung carcinomas, when compared with normal lung tissue, suggesting that *MEAK7* may play a role in lung tumorigenesis (Figures 1D and 1E) (Rhodes et al., 2004). Also, an association was found between *MEAK7* expression and outcomes of patients with cancer. Patients who died from ductal breast carcinoma (p = 2.72 × 10^−6^, Fold Change: 2.136) and acute myeloid leukemia (p = 7.99 × 10^−5,^ Fold Change: 2.655) had enhanced *MEAK7* expression (Figures S1B and S1C) (Rhodes et al., 2004). Thus the *MEAK7* expression profile of patients with cancer may provide insight into predicting patient prognosis and survival.

**Figure 2:**
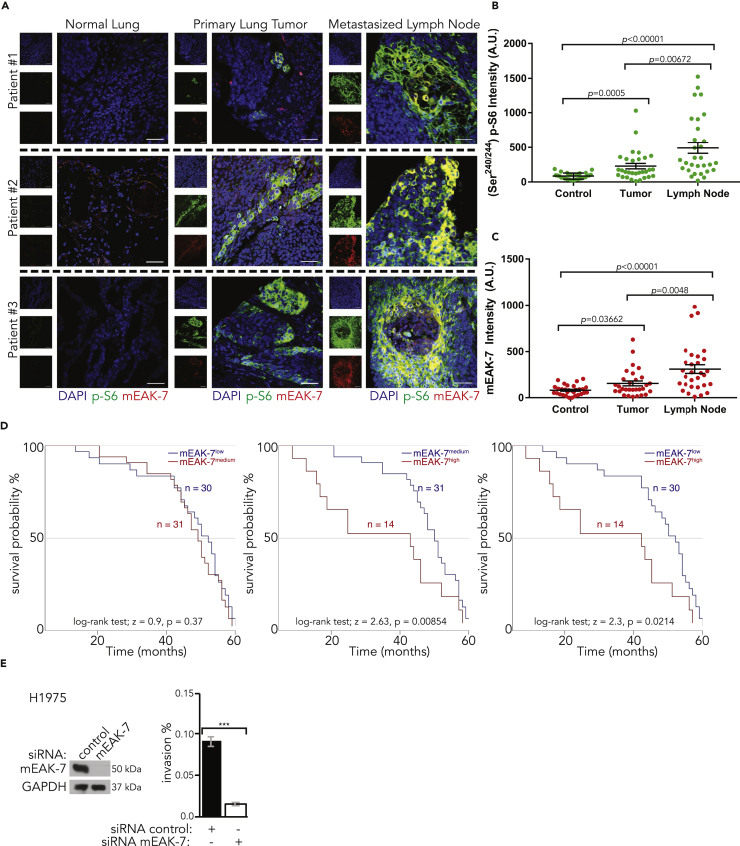
mEAK-7 Protein Levels Are High in Nearby Lymph Nodes of the Tumor Mass in Patients with NSCLC (A) Three representative human patient tissue sections stained for (Ser^240/244^) p-S6 and mEAK-7. Scale bar, 250 μm. (B) Statistical analysis of 30 paired patients with NSCLC with the normal lung, primary tumor, and metastasized lymph node for (Ser^240/244^) p-S6 staining. Mann-Whitney U test was used. (C) Statistical analysis of 30 paired patients with NSCLC with the normal lung, primary tumor, and metastasized lymph node for mEAK-7 staining. Mann-Whitney U test was used. (D) NSCLC tissue microarray analysis of Kaplan-Meier survival curves. Log rank test was used. (E) H1975 cells were treated with control or mEAK-7 siRNA for 48 h; 50,000 cells were seeded into *in vitro* Matrigel-based invasion chambers and allowed to grow for 24 h. Analyzed by Student's t test (n = 6). ***p < 0.0001. Experiments were repeated at least six times.

In a screen of human squamous cell carcinomas, the UM-SCC-17A cell line (Brenner et al., 2010), derived from the primary laryngeal cancer site of a 48-year-old female patient, did not express detectable levels of mEAK-7 protein (Nguyen et al., 2018). Interestingly, the UM-SCC-17B cell line, derived from a metastatic site from the same patient, did express mEAK-7 (Nguyen et al., 2018). These findings suggest that increased expression of mEAK-7 may be associated with tumor metastasis. To test the hypothesis that elevated mEAK-7 protein levels are associated with cancer metastasis, the protein expression patterns of primary tumors and confirmed metastasizes closest to lymph tissues of patients with cancer were compared with healthy lung and lymph tissues. An NSCLC tissue microarray containing 30 individual pathologist-graded, patient-matched sections of primary tumors, as well as their normal adjacent tissue, and metastatic lymph nodes were stained and analyzed. mEAK-7 and (Ser^240/244^) p-S6 protein levels were significantly elevated in the primary human tumor when compared with the normal adjacent tissue (Figures 2A–2C). Furthermore, mEAK-7 and (Ser^240/244^) p-S6 protein levels were significantly greater in the metastatic lymph nodes when compared with both primary tumor and normal adjacent tissue (Figures 2A–2C). However, healthy adult lymph tissue did not yield substantial mEAK-7 or (Ser^240/244^) p-S6 protein levels (Figure S2A). Thus, mEAK-7 protein and activated mTOR signaling was found to be enhanced in primary NSCLC, and substantially detected in metastasized lymph nodes, suggesting that mEAK-7 could function as a biomarker for patients with metastasized cancers.

As Oncomine databases demonstrate that higher *MEAK7* expression is associated with poor patient prognoses (Figures S1B and S1C), we sought to investigate this via a lung adenocarcinoma tissue microarray detailing patient survival data. The microarray stained with antibodies targeting endogenous mEAK-7 and (Ser^240/244^) p-S6 and a Kaplan-Meier Survival Curve analysis illustrated that mEAK-7 protein levels were strongly associated with poor patient prognosis, specifically in patients with relapse following surgical intervention (Figure 2D).

Because mEAK-7 protein levels were highly expressed in metastasized lymph nodes, we tested the extent to which mEAK-7 is required for cell invasion *in vitro*. To test this, H1975 cells were treated with control or mEAK-7 small interfering RNA (siRNA) for 48 h and 50,000 cells were seeded into invasion chambers for 24 h. Results demonstrated a statistically significant reduction in cell invasion after 24 h (Figure 2E). Thus, mEAK-7 may be a clinically relevant biomarker for predicting the prognosis of patients with metastatic NSCLC.

### Cancer Stem Cells Exhibit High Protein Levels of mEAK-7 and mTOR Signaling

**Figure 3:**
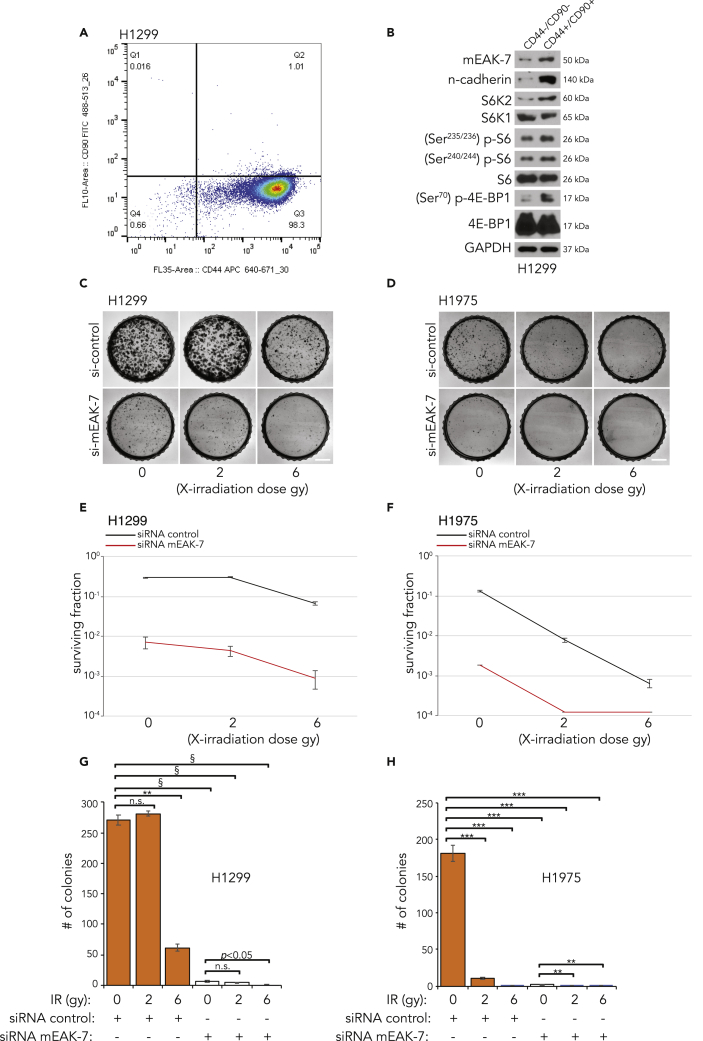
mEAK-7 Is Expressed in CD44+/CD90+ Group and Required for Clonogenic Potential and Radiation Resistance (A) Flow sort diagram depicting the CD44+/CD90+ cell population in H1299 cells. (B) Immunoblot analysis of CD44−/CD90− and CD44+/CD90+ H1299 cells for mEAK-7 and mTOR signaling. (C) H1299 cells were treated with control or mEAK-7 siRNA for 48 h, X-ray irradiated at 2 or 6 Gy, and 2,500 cells were seeded into 60-mm tissue culture plates (TCPs) and grown for 10 days. (D) H1975 cells were treated with control or mEAK-7 siRNA for 48 h and subjected to no treatment or 2- and 6-Gy X-ray irradiation, and 5,000 cells were seeded into 60-mm TCPs and grown for 10 days. Scale bars, 2.5 mm. (E) H1299 surviving fraction analysis for (C). (F) H1975 surviving fraction analysis for (D). (G) H1299 colony number graphs for (C). (H) H1975 colony number graphs for (D). Analysis of clonogenic assay via Student's t test. **p < 0.001, ***p < 0.0001, ^§^p < 0.000001. All experiments were repeated at least three times, and (C–H) was repeated six times.

mEAK-7 is a strong, positive regulator of mTOR signaling (Nguyen et al., 2018), and several groups have demonstrated that mTOR signaling is essential for CSC self-renewal (Matsubara et al., 2013) and radiation resistance (Fang et al., 2013). Therefore, we hypothesized that mEAK-7 is differentially expressed in the CSC versus non-CSC populations. Because the majority of information on human mEAK-7 has been demonstrated in NSCLC cell lines H1975 and H1299, we sought to determine the expression profile of mEAK-7 and mTOR signaling in NSCLC CSCs. These cells are best identified as being CD44+ (Leung et al., 2010) and CD90+ (Wang et al., 2013). Therefore, H1299 cells were subjected to fluorescence-activated cell sorting for the aforementioned markers. CD44+/CD90+ cells, representing 1% of all cells sorted, were analyzed for mEAK-7 and mTOR signaling (Figure 3A). Immunoblot analysis demonstrated that CD44+/CD90+ H1299 cells, when compared CD44−/CD90−, yielded greater protein levels of mEAK-7 and S6K2, as well as activated mTOR signaling, as measured via p-S6 and p-4E-BP1 protein levels (Figure 3B). The CD44+/CD90+ cell population expressed n-cadherin (Figure 3B), which is known as an essential component of the epithelial-mesenchymal transition state in CSCs and suggests that high levels of mEAK-7 in cancer cells are more likely to metastasize (Liu et al., 2014). Similar results were observed in CD44+/CD90+ H1975 cells (Figures S2B and S2C). Collectively, these results demonstrate that there is a selective expression profile of mEAK-7 and S6K2 in self-renewing CSCs.

### mEAK-7 Is Necessary for Clonogenic Potential and Spheroid Formation in Human Cancer Cells

To test the hypothesis that mEAK-7 affects cell survival following X-ray irradiation damage, a 2D clonogenicity assay was used to assess the surviving fraction of irradiated cancer cells (Franken et al., 2006, Puck and Marcus, 1956). H1299 and H1975 cells were treated with control or mEAK-7 siRNA for 48 h and then subjected to no treatment or X-ray irradiation of 2 or 6 Gy. These cells were then reseeded into new 60-mm tissue culture plates and allowed to grow for 10 days. In both H1299 and H1975 cells, clonogenic potential was significantly decreased after mEAK-7 knockdown, but little additional effects were found after exposure to 2- and 6-Gy X-ray irradiation in both H1299 and H1975 cells (Figures 3C–3H). This suggests that mEAK-7 alone is capable of regulating clonogenic potential to a dramatic extent. In H1299 and H1975 cells, similar results were observed at higher cell seeding densities (Figure S2D) or by utilizing a different siRNA mEAK-7 (Figures S3A and S3B), demonstrating that mEAK-7 enhances the clonogenic potential of human cancer cells in response to DNA damage.

**Figure 4:**
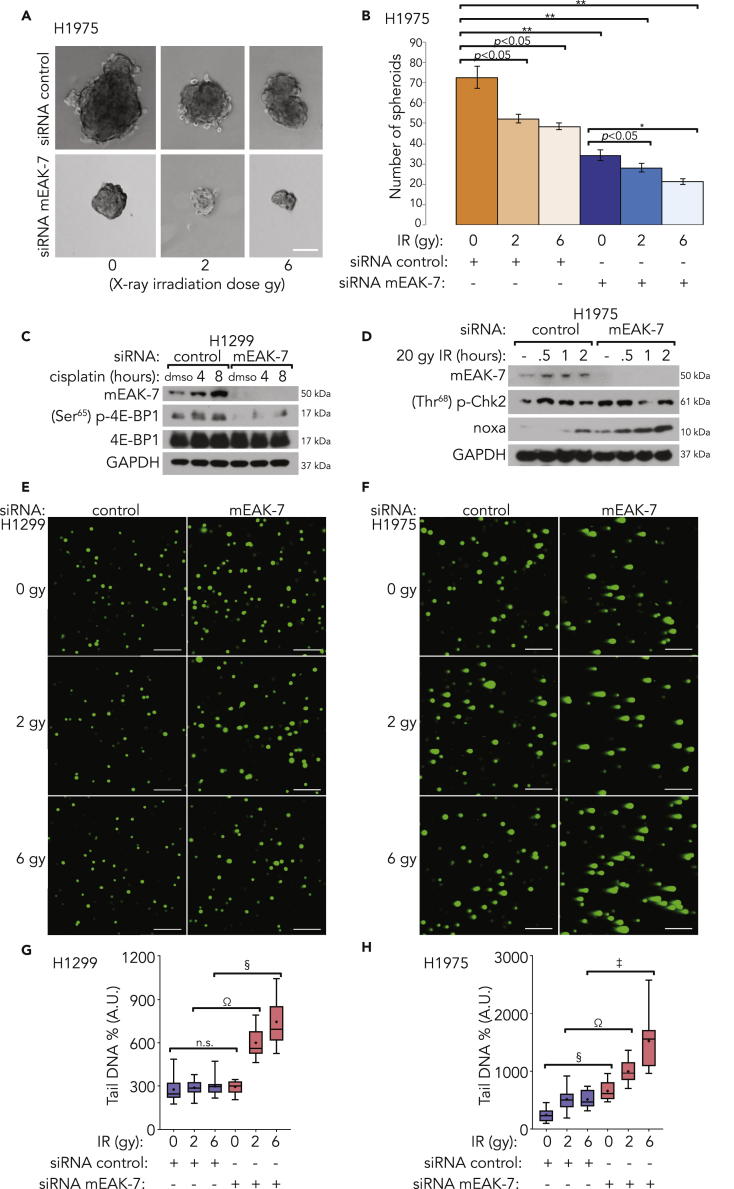
mEAK-7 Is Required for Spheroid Formation and Is Necessary for an Effective DNA Damage Response (A) Images of spheroids. H1975 cells were treated with control or mEAK-7 siRNA, X-ray irradiated at 2 or 6 Gy, and 10,000 cells were seeded into 60-mm ultra-low attachment plates and grown for 10 days. Scale bar, 125 μm. (B) Quantification of spheroid formation and analysis via Student's t test (n = 6) of (A). *p < 0.01, **p < 0.001. (C) H1299 cells were treated with control or mEAK-7 siRNA and with DMSO or 10 μM cisplatin for 4 or 8 h, and mTOR signaling was analyzed. (D) H1975 cells were treated with control or mEAK-7 siRNA and X-ray irradiated at 20 Gy for 30 min, 1 h, or 2 h and analyzed for Noxa expression by DNA damage response. (E and F) H1299 and H1975 cells were treated with control or mEAK-7 siRNA and X-ray irradiated at 2 or 6 Gy for 30 min and assessed via the comet assay to detect damaged DNA. Scale bar, 250 μm. (G and H) Statistical analysis via Student's t test (n = 15) of (E and F) represented as box plots. *p < 0.01, **p < 0.001, ^‡^p < 0.00001, ^§^p < 0.000001, ^Ω^p<0.0000001. All experiments were repeated at least three times.

Two-dimensional assays measuring clonogenic potential demonstrate the essential role of mEAK-7 in the DNA damage response, whereas 3D assays better simulate *in vivo* conditions. The spheroid-forming assay is a widely accepted experimental strategy to identify stem cell self-renewal in mammalian cell systems *in vitro* and *in vivo* (Pastrana et al., 2011). Furthermore, CSCs are the principal cancer cell population responsible for spheroid formation (Weiswald et al., 2015). Thus, to test the effect of mEAK-7 on spheroid size and formation, H1975 cells were treated with control or mEAK-7 siRNA for 48 h, subjected to no treatment or 2- or 6-Gy X-ray irradiation, and subsequently seeded in ultra-low attachment dishes for 1 week. mEAK-7 knockdown resulted in a dramatic reduction in spheroid size with no treatment, or 2- or 6-Gy treatment (Figure 4A). In addition, the gross number of spheroids formed was significantly reduced after mEAK-7 knockdown, and even further reduced following 2- or 6-Gy X-ray irradiation treatment (Figure 4B). Similar results were observed when H1975 cells were seeded at a lower density (Figure S3C) and when a different mEAK-7 siRNA was used in both H1299 and H1975 cells (Figures S3D and S3E). These results collectively indicate that mEAK-7 plays a significant role in cancer cell spheroid formation.

### mEAK-7 Is Necessary for Chemoresistance, Radiation Resistance, and Sustained DNA Damage-Mediated mTOR Signaling in Human Cancer Cells

Although X-ray irradiation is a potent inducer of the DNA damage response pathway, we hypothesized that other forms of genotoxic stress may similarly regulate mEAK-7. Cisplatin is a chemotherapeutic drug often used to treat patients with cancer with solid tumors. Cisplatin is widely used for the treatment of solid tumors because it has the ability to activate the DNA damage response and allow cells to undergo apoptosis. Although the mechanism of action would take 20 years to unravel, it was clear that cisplatin produced DNA adducts and forced double-stranded breaks in the DNA. However, following initial tumor regression, a fraction of solid tumors become chemoresistant (Galluzzi et al., 2012). mTOR signaling can be activated during cisplatin treatment of ovarian cancer cells over time, as measured by enhanced (Thr^389^) p-S6K1 protein levels (Peng et al., 2010). As mEAK-7 is required for mTOR signaling, we tested the hypothesis that mEAK-7 is an essential modulator of mTOR signaling following genotoxic stress induced by cisplatin treatment. To test the extent to which genotoxic stress modifies mEAK-7 activity, H1299 cells were treated with mEAK-7 siRNA for 48 h. Next, these cells were treated with either DMSO or 10 μM cisplatin for 4 or 8 h. mEAK-7 protein levels were increased in response to DNA damage by cisplatin treatment. Furthermore, mEAK-7 was required for regulation of (Ser^65^) p-4E-BP1, an indicator of mTOR signaling (Figure 4C). Therefore, cisplatin is capable of regulating mEAK-7-mediated mTOR signaling.

### mEAK-7 Knockdown Impairs the DNA Damage Response and Enhances Noxa Levels after X-Ray Irradiation

Cancer cells utilize many mechanisms to promote radioresistance in response to cancer therapies (Kim et al., 2009). To determine the role of X-ray irradiation on mEAK-7 and the DNA damage response, H1975 cells were treated with control or mEAK-7 siRNA, and X-ray irradiated with 20 Gy for up to 2 h. H1975 cells treated with mEAK-7 siRNA exhibited a dramatic increase in Noxa levels (Figure 4D). We also found similar results in H1299 cells treated with mEAK-7 siRNA and subjected to X-ray irradiation (Figure S3F). Upregulation of Noxa occurs in response to DNA damage after mEAK-7 knockdown (Figure 4D), which ultimately leads to cellular apoptosis (Ploner et al., 2008), suggesting that mEAK-7 plays a role in the DNA damage response and cancer cell survival. Noxa is known to be regulated by p53-dependent mechanisms through oncogene-independent apoptosis induced by genotoxic stressors, where p53 activates both survival and apoptotic pathways through p21WAF1/Cip1 (Shibue et al., 2003). However, Noxa has also been shown to function through p53-independent mechanisms, where ATF3 and ATF4 are induced by cisplatin, a DNA-damaging agent, and downregulation of ATF3 or ATF4 reduces Noxa expression because ATF3 and ATF4 bind to and cooperatively activate the Noxa promoter (Sharma et al., 2018). As H1975 is a p53 wild-type NSCLC line and H1299 is a p53-null NSCLC line, mEAK-7 may regulate Noxa expression through a p53-independent mechanism.

The comet assay is used to quantify intracellular DNA damage resulting from an insufficient DNA damage repair response in eukaryotic cells (Collins, 2004). H1299 and H1975 cells were independently embedded in an agarose gel and lysed, thereby releasing intracellular DNA. During gel electrophoresis, DNA fragments migrate toward the anode, resulting in a “comet” pattern with a trail of DNA fragments, such that a longer trail signifies more fragments, and therefore more DNA damage (Ostling and Johanson, 1984). Owing to the dramatic results from radiation treatment with mEAK-7 knockdown in the clonogenicity (Figures 3C–3H) and spheroid assays (Figures 4A and 4B), we hypothesized that mEAK-7 plays a crucial role in the DNA damage repair pathways. The visualization of DNA strand migration via the comet assay, and subsequent quantification of that migration, allowed us to predict the relative levels of the DNA repair response. H1299 and H1975 cells were treated with control or mEAK-7 siRNA and subjected to no treatment or 2- or 6-Gy X-ray irradiation for 30 min. Comet pattern formations, quantified as tail DNA %, were enhanced in H1299 and H1975 cells treated with mEAK-7 siRNA and X-ray irradiation (Figures 4E–4H), suggesting mEAK-7 is necessary for the DNA repair response in these cancer cells.

### mEAK-7 Interacts with DNA-PKcs in Response to X-Ray Irradiation Damage

**Figure 5:**
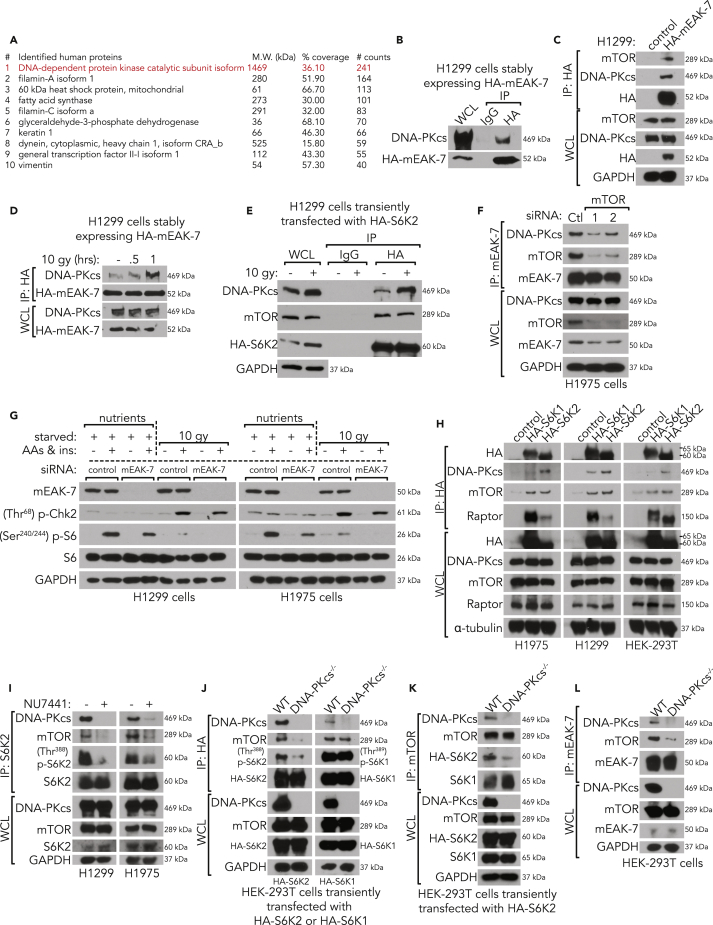
mEAK-7 Forms a Third mTOR Complex with DNA-PKcs to Regulate S6K2 Activity (A) H1299 cells stably expressing HA-mEAK-7 were lysed in 3-((3-cholamidopropyl) dimethylammonio)-1-propanesulfonate (CHAPS) buffer, HA-mEAK-7 was immunoprecipitated, and co-immunoprecipitated proteins were analyzed for mass spectrometry quantitative profiling. (B) H1299 cells stably expressing HA-mEAK-7 were lysed in NP40 lysis buffer and using IgG control antibody or HA-tag antibody, HA-mEAK-7 was immunoprecipitated to check DNA-PKcs interaction. (C) Non-transduced H1299 cells or H1299 cells stably expressing HA-mEAK-7 were lysed in CHAPS lysis buffer, and HA-tag antibody was used for immunoprecipitation to check DNA-PKcs or mTOR interaction. (D) H1299 cells stably expressing HA-mEAK-7 were untreated or X-ray irradiated at 10 Gy for 30 min and 1 h and lysed in NP40 lysis buffer. HA-mEAK-7 was immunoprecipitated to check DNA-PKcs interaction. (E) H1299 cells were transiently transfected with pcDNA3-HA-S6K2 and then untreated or X-ray irradiated at 10 Gy for 1 h. HA-S6K2 was immunoprecipitated to check DNA-PKcs or mTOR interaction. (F) H1975 cells were transiently transfected with control or mTOR #1 or mTOR #2 siRNA for 48 h. Cells were collected in CHAPS, and endogenous mEAK-7 was immunoprecipitated to check DNA-PKcs interaction. (G) H1299 and H1975 cells were transiently transfected with control or mEAK-7 siRNA for 48 h. Cells were subsequently starved of nutrients for 1 h and replenished with DMEM^+AAs^ and 10 μM insulin for 30 min or cultured normally and treated with 10-Gy X-ray irradiation for 30 min. Immunoblot analysis was conducted for mTOR signaling proteins. (H) H1975, H1299, and HEK293T cells were transfected with no plasmid, pRK7-HA-S6K1-WT, and pcDNA3-HA-S6K2. HA-tag antibody was immunoprecipitated and probed for DNA-PKcs, mTOR, and Raptor. (I) H1299 and H1975 cells were treated with either DMSO or NU7441 (DNA-PKcs inhibitor) at 5 μM for 2 h and mixed in fresh DMEM with 10% FBS. Cells were collected in CHAPS, S6K2 was immunoprecipitated, and immunoblots were utilized to assess mTOR signaling. GAPDH was used for loading controls. (J) DNA-PKcs^WT^ and DNA-PKcs^−/−^ HEK293T cells were transfected with pcDNA3-HA-S6K2 or pRK7-HA-S6K1-WT. Before protein isolation, cells were fed fresh DMEM with 10% FBS for 1 h before collection in CHAPS. HA-tag antibody was immunoprecipitated and probed for mTOR and activated S6K2 signaling. (K) Experimental protocols were repeated from (J), and mTOR antibody was immunoprecipitated and probed for mTOR, HA-S6K2, and S6K1. (L) DNA-PKcs^WT^ and DNA-PKcs^−/−^ HEK-293T cells were collected in CHAPS lysis buffer. Endogenous mEAK-7 was immunoprecipitated and probed for mTOR and DNA-PKcs. All experiments were repeated at least three times.

To determine the extent to which mEAK-7 regulates genotoxic activation of mTOR signaling, we sought to identify interacting partners of mEAK-7 by transducing H1299 cells with a lentivirus expressing pLenti-GIII-HA(c-term)mEAK-7. Through hemagglutinin (HA)-mEAK-7 immunoprecipitation (IP) and mass spectrometric (MS) analysis, a list of proteins that potentially interact with mEAK-7 was generated (Table S1). DNA-dependent protein kinase catalytic subunit isoform 1 (DNA-PKcs) scored the highest with 241 exclusive spectral counts and 36% protein coverage (Figure 5A). As with the canonical role of DNA-PKcs in the DNA damage response, ataxia mutated (ATM) and ataxia telangiectasia- and Rad3-related (ATR) also play essential and redundant roles in repairing damaged DNA, because all three share similar targets in response to DNA damage (Ciccia and Elledge, 2010, Nguyen et al., 2018). These data suggest that other phosphatidylinositol 3-kinase-related kinase (PIKK) members, like DNA-PKcs and mTOR, could also work in concert to regulate metabolism. Thus, we posited that mEAK-7 regulates mTOR signaling, in part, through the interaction with DNA-PKcs.

To confirm our IP/MS results, protein lysates were harvested from H1299 cells stably transduced with HA-mEAK-7. Exogenous mEAK-7 interacted with endogenous DNA-PKcs (Figures 5B and 5C). Under UV B irradiation, DNA-PKcs has been shown to interact with mTOR kinase and SIN1 to affect mTORC2 signaling in epithelial skin keratinocytes, but the molecular rationale for this interaction remains elusive (Tu et al., 2013). Furthermore, nuclear DNA-PKcs transverses to the cytosol in response to DNA damage (Tu et al., 2013). This ability to travel to the cytoplasm, combined with evidence that DNA-PKcs can activate metabolism-related genes, suggests a role for DNA-PKcs in metabolic signaling.

Because mEAK-7 interacts with mTOR in increasing amounts of amino acid, insulin, or nutrient conditions (Nguyen et al., 2018), we sought to determine the extent to which mEAK-7 increases its binding to DNA-PKcs after DNA damage. H1299 cells stably expressing HA-mEAK-7 were subjected to no treatment or X-ray irradiated with 10 Gy for 30 or 60 min, and results revealed that mEAK-7 increasingly interacted with DNA-PKcs over time in response to DNA damage (Figure 5D). These data suggest that mEAK-7 associates with DNA-PKcs and mTOR to form a third mTOR complex (mTORC3), distinct from mTORC1 and mTORC2.

First, to determine the extent to which S6K2 is required for mTOR signaling following X-ray irradiation, H1975 and H1299 cells were treated with controls or two unique S6K1 or S6K2 siRNAs. Results revealed that p-S6 levels were abrogated after S6K2 knockdown in H1975 and H1299 cells (Figures S4A and S4B), suggesting that S6K2 is necessary for sustained S6 phosphorylation in response to DNA damage. S6K2 is capable of binding to mTOR in the presence of nutrient stimulation (Nguyen et al., 2018), but it is unknown whether S6K2 binds to DNA-PKcs. As mEAK-7 is required for the mTOR-S6K2 axis (Nguyen et al., 2018), DNA-PKcs and S6K2 binding was tested to determine the extent to which mEAK-7 and DNA-PKcs form a complex that regulates mTOR signaling through S6K2. To test whether S6K2 interacts with DNA-PKcs in the presence of DNA damage, H1299 cells were transiently transfected with pcDNA3-HA-S6K2-WT; subsequently, they were X-ray irradiated with 10 Gy for 1 h and HA-S6K2 was immunoprecipitated. X-ray irradiation considerably increased the interaction between DNA-PKcs and HA-S6K2, whereas it had little to no influence on mTOR and HA-S6K2 interaction (Figure 5E). Conversely, HA-S6K1 binding to DNA-PKcs was observed, but there was a severe reduction in binding after DNA damage, suggesting that S6K1 is not the main downstream target of DNA-PKcs after DNA damage (Figure S4C). These findings were reproduced under nutrient stimulation as well, where DNA-PKcs enhances binding to HA-S6K2 and DNA-PKcs diminishes binding to HA-S6K1 (Figure S4D). DNA-PKcs is capable of interacting with S6K2 to regulate its function in response to DNA damage. To determine if mTOR is required for mEAK-7-mediated DNA-PKcs function and interaction, mTOR was knocked down with two different mTOR siRNAs, which resulted in a dramatic reduction of DNA-PKcs interaction with endogenous mEAK-7 (Figure 5F). This collective evidence links mEAK-7 to the major metabolic sensor, mTOR, and the crucial DNA damage repair regulator, DNA-PKcs.

Although we previously established the function of mEAK-7 in mTOR signaling under nutrient conditions (Nguyen et al., 2018), its role in mTOR signaling under genotoxic stressors remains unknown. To test the hypothesis that mEAK-7 is required for mTOR activation after DNA damage, H1299 and H1975 cells were treated with control or mEAK-7 siRNA for 48 h in DMEM with 10% fetal bovine serum (FBS). Two different conditions were compared: 1-h nutrient starvation with 30-min nutrient replenishment was compared with 10-Gy X-irradiation for 30 min. Under these conditions, mEAK-7 was also capable of regulating sustained activation of mTOR signaling after X-ray irradiation in NSCLC (Figure 5G). Thus, mEAK-7 is capable of regulating nutrient-dependent mTOR signaling, as well as X-ray irradiated activation of mTOR signaling.

Next, to determine the relative binding affinities of S6K1 and S6K2 to their most relevant complex, we transfected H1299, H1975, and HEK-293T cells with HA-S6K1 or HA-S6K2 and immunoprecipitated HA-S6K1 or HA-S6K2 to probe for mTOR, raptor, and DNA-PKcs binding. Interestingly, we discovered that HA-S6K1 is capable of binding to DNA-PKcs, but HA-S6K2 binds to DNA-PKcs to a significantly larger extent (Figure 5H). Furthermore, we found that raptor binding to HA-S6K1 was strong, whereas HA-S6K2 binding to raptor was weaker (Figure 5H). This suggests that S6K1 and S6K2 have preferential binding affinities to mTORC1, or alternative mTOR complexes. Finally, to test the extent to which DNA-PKcs activity is required for S6K2 function, H1299 and H1975 cells were treated with either DMSO or 5 μM NU7441, a highly specific chemical inhibitor of DNA-PKcs, for 2 h under freshly stimulated 10% FBS medium. NU7441 is 1,000× more specific for DNA-PKcs than PI3K and 200× more specific for DNA-PKcs than mTOR. DNA-PKcs inhibition resulted in a substantial decrease in S6K2/mTOR/DNA-PKcs binding, as well as S6K2 functional activity (Figure 5I). These results suggest that mEAK-7 structurally links DNA-PKcs and S6K2 to mTOR signaling.

Although pharmacologic agents are potent molecules to dissect the downstream pathways within cells, they still may yield non-specific targeting. To rule out non-specific interactions of DNA-PKcs inhibitors with other kinases, we obtained validated DNA-PKcs^−/−^ HEK293T cells (Neal et al., 2016). In DNA-PKcs^−/−^ cells, IP demonstrated that HA-S6K2 phosphorylation was diminished and HA-S6K2 binding to mTOR was reduced, but HA-S6K1 phosphorylation and HA-S6K1 binding to mTOR were minimally affected (Figure 5J). IP of mTOR in DNA-PKcs^−/−^ cells transfected with HA-S6K2 resulted in abrogated binding to HA-S6K2 and increased binding to HA-S6K1, demonstrating that mTOR utilizes DNA-PKcs to form a new complex to target S6K2 (Figure 5K). Finally, loss of DNA-PKcs inhibited the ability of mEAK-7 to bind to mTOR, suggesting that mEAK-7, DNA-PKcs, and mTOR form a stable complex to regulate S6K2 (Figure 5L).

### mEAK-7 Is Required for Sustained IR-Mediated mTOR Signaling in Human Cancer Cells and Loss of mEAK-7 Results in Enhanced PARP Cleavage

**Figure 6:**
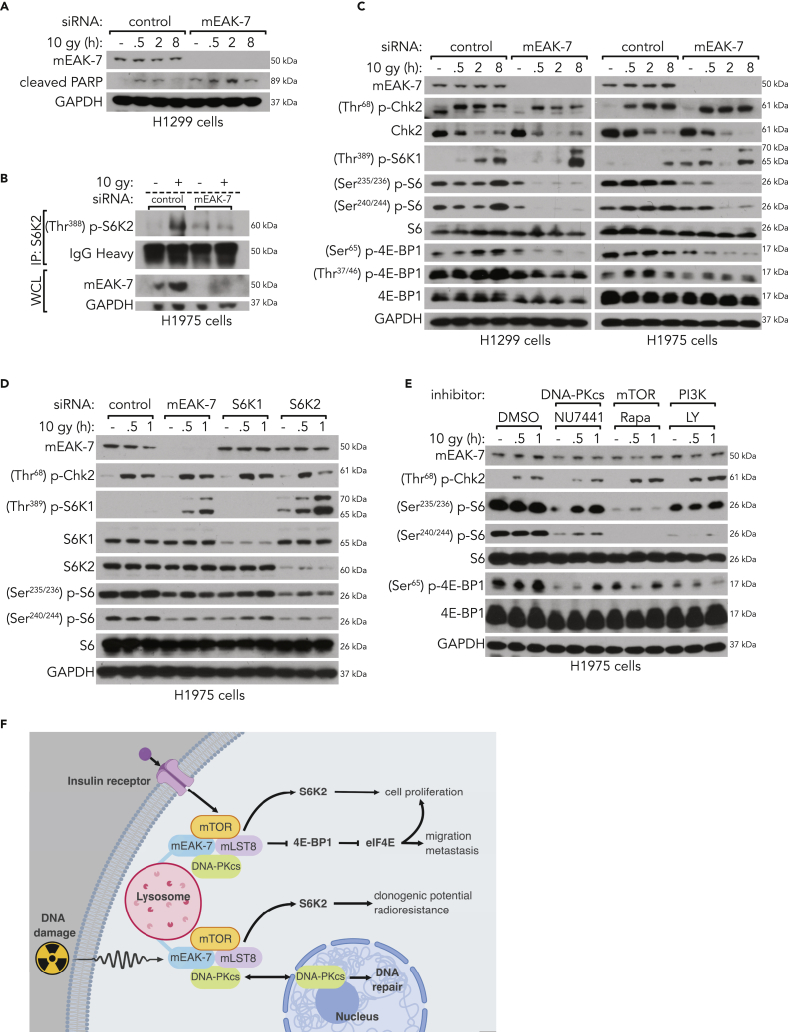
mEAK-7 and DNA-PKcs Are Required for X-Ray Irradiation-Mediated mTOR Signaling (A) H1299 cells were treated with control or mEAK-7 siRNA for 48 h; X-ray irradiated at 10 Gy for 30 min, 2 h, and 8 h; and analyzed for PARP cleavage. (B) H1975 cells were transiently transfected with control or mEAK-7 siRNA for 48 h. Next, cells were treated with 10-Gy X-ray irradiation for 30 min, followed by IP of endogenous S6K2, and probed for activated S6K2 signaling. (C) H1299 and H1975 cells were treated with control or mEAK-7 siRNA for 48 h; X-ray irradiated at 10 Gy for 30 min, 2 h, and 8 h; and analyzed for mTOR signaling. (D) H1975 cells were treated with control, mEAK-7, S6K1, and S6K2 siRNA for 48 h; X-ray irradiated at 10 Gy for 30 min and 1 h; and analyzed for mTOR signaling. (E) H1975 cells were treated with DMSO, DNA-PKcs inhibitor (5 μM NU7441 IC50 = 14 nM), mTOR inhibitor (100 nM rapamycin, IC50 = 1 nM), and PI3K inhibitor (50 μM LY249002, IC50 = 2.3 μM) for 1 h before being treated with X-ray irradiation at 10 Gy for 30 min and 1 h and analyzed for mTOR signaling. (F) Working model for a mEAK-7-mTOR-DNA-PKcs complex. All experiments were repeated at least three times. GAPDH was used for loading controls.

As we determined that mEAK-7 knockdown combined with X-ray irradiation resulted in strong Noxa upregulation, we posited that other markers of cell apoptosis would also be affected by mEAK-7. PARP cleavage has been shown to be an essential mediator of cell apoptosis and cell death (Morales et al., 2014). To determine the extent to which mEAK-7 regulates PARP cleavage, H1299 cells treated with mEAK-7 siRNA resulted in enhanced levels of cleaved PARP (Figure 6A). These results suggest mEAK-7 is required for radiation resistance and that the loss of mEAK-7 results in severe reduction of mTOR signaling and enhanced PARP cleavage.

Next, we posited that mEAK-7 was required for S6K2 phosphorylation and activation after X-ray irradiation. H1975 cells were treated with control or mEAK-7 siRNA for 48 h and 10-Gy X-ray irradiation for 30 min. IP of endogenous S6K2 demonstrated that mEAK-7 knockdown resulted in a dramatic decrease in X-ray irradiation-mediated S6K2 phosphorylation (Figure 6B). Use of a different mEAK-7 siRNA also resulted in a decrease in S6K2 phosphorylation after DNA damage (Figure S4E). Moreover, the literature details examples of sustained mTOR signaling following DNA damage as a modulator of self-renewal and radiation resistance (Silvera et al., 2017). To test the hypothesis that mEAK-7 is necessary for sustained X-ray irradiation-mediated mTOR signaling, H1299 and H1975 cells were treated with control or mEAK-7 siRNA for 48 h in DMEM with 10% FBS and then with 10-Gy X-irradiation for 30 min, 2 h, or 8 h. H1299 and H1975 cells treated with mEAK-7 siRNA exhibited abrogated mTOR signaling over time (Figure 6C). These results were also confirmed in MDA-MB-231 cells, a triple-negative breast carcinoma cell line (Figure S5). Therefore, these results suggest that mEAK-7 is required for sustained mTOR signaling as well as cell survival after DNA damage, and that the loss of mEAK-7 results in severe reduction of mTOR signaling and enhanced PARP cleavage.

Both S6K1 and S6K2 are essential components of mTOR signaling that are described to have similar, but distinct, cellular roles in human development and disease (Pardo and Seckl, 2013). To elucidate the roles of S6K1 and S6K2 following X-ray irradiation damage, (Ser^240/244^) p-S6 levels were measured in response to siRNA-mediated knockdown of mEAK-7, S6K1, or S6K2 and treatment with 10-Gy X-ray irradiation. H1975 cells were treated with control, mEAK-7, S6K1, or S6K2 siRNA for 48 h in DMEM with 10% FBS. Subsequently, the cells were treated with 10-Gy X-ray irradiation for 30 min or 1 h. mEAK-7 and S6K2 knockdown each markedly reduced (Ser^240/244^) p-S6 levels, but S6K1 knockdown did not have a substantial effect on (Ser^240/244^) p-S6 levels (Figure 6D). These results mirrored the nutrient conditions, as previously published (Nguyen et al., 2018). Furthermore, mEAK-7 or S6K2 knockdown dramatically increased (Thr^389^) p-S6K1 levels following X-ray irradiation-induced damage, suggesting a specific role for S6K2 and mEAK-7 during X-ray irradiation-mediated mTOR signaling (Figure 6D).

Although some reports suggest a possible intersection of DNA-PKcs and mTOR signaling, these ideas have not yet been fully validated across a spectrum of cell types and experimental conditions. In an effort to test the hypothesis that DNA-PKcs and mTOR signaling depend on mEAK-7 to carry out a shared function, we treated H1299 and H1975 cells with NU7441. NU7441 treatment significantly reduced infrared (IR)-mediated mTOR signaling in a dose-dependent manner, but had little effect on (Ser^2448^) p-mTOR levels (Figure S6A). To determine whether NU7441 significantly inhibits IR-induced activation of mTOR signaling compared with other mTOR inhibitors, we used specific inhibitors of DNA-PKcs (NU7441), mTOR (rapamycin), and PI3K (LY249002). Inhibition of DNA-PKcs, mTOR, or PI3K significantly decreased mTOR signaling in H1975 cells (Figure 6E). These results were also consistent in H1299 cells (Figure S6B). In conclusion, DNA-PKcs is a fundamental component of a novel mTOR complex that regulates the mEAK-7/mTOR signaling axis and targets S6K2.

## Discussion

Surgical intervention and radiation therapy are common treatment modalities for patients with solid tumors. However, in many patients the condition relapses as tumors acquire resistance through intratumoral evolution (McGranahan and Swanton, 2017). CD44+/CD90+ cells have been identified as a unique population of cancer cells that may be required for the regulation of chemo- and radioresistance through PI3K and mTOR signaling (Chang et al., 2013). Furthermore, the literature demonstrates that S6K2 inhibits apoptosis in lung cancer (Pardo et al., 2006) and that S6K2 amplification is associated with more aggressive forms of breast cancer (Pérez-Tenorio et al., 2011). Similarly, a retrospective study conducted on patients with breast cancer demonstrated that 4E-BP1 and S6K2 were correlated with poor prognosis and endocrine resistance (Karlsson et al., 2013). This evidence, combined with our findings that mTOR signaling, mEAK-7, and S6K2 are upregulated in CD44+/CD90+ cancer cell populations, suggests that mEAK-7 is involved in mTOR signaling in CSCs.

Although CD44+/CD90+ cells demonstrate radiation resistance and self-renewal capacity that correlate with elevated mEAK-7 protein levels, loss of mEAK-7 alone does not result in enhanced cell apoptosis in human cancer cells (Nguyen et al., 2018). The combination of DNA damage and loss of mEAK-7 is capable of enhancing cell apoptosis. This is likely because the mechanisms that support cell survival and self-renewal are different. As PI3K and mTOR signaling are crucial regulators of radiation resistance and self-renewal in many carcinomas, including cervical carcinoma (Kim et al., 2010), head and neck squamous cell carcinoma (Leiker et al., 2015), and breast carcinoma (Steelman et al., 2011), it is important to note that mEAK-7 is also a strong effector of these processes. Specifically, CSCs have been identified as a cell population that modulates radiation resistance and self-renewal in solid tumors (Pajonk et al., 2010).

Alternative mTOR signaling appears to be upregulated in human patients with cancer, specifically in patients with metastatic disease (Figures 2A–2C). Here, DNA-PKcs was identified as a new interacting partner of mEAK-7 (Figures 5A–5H) and may participate in the regulation of this alternative mTOR signaling. DNA-PKcs is a member of the PIKK family that includes mTOR, ATM, ATR, suppressor of morphogenesis in genitalia (SMG1), and transformation/transcription domain-associated protein (TRRAP) (Lovejoy and Cortez, 2009). DNA-PKcs has been extensively studied in the context of non-homologous end joining and homologous recombination, both of which are DNA damage repair pathways (Smith and Jackson, 1999). PIKKs typically have redundant cellular roles, depending on their cellular localization and biologic context. For example, DNA-PKcs, ATM, and ATR all have similar cellular targets in response to DNA damage (Ciccia and Elledge, 2010). Intriguingly, DNA-PKcs was found to play a critical role in metabolic gene regulation in response to insulin (Wong et al., 2009). However, DNA-PKcs predominantly resides in the nucleus, so it was initially unclear how DNA-PKcs could exit the nucleus to affect nutrient metabolism. The literature supports the observation that pockets of DNA-PKcs exists in lipid rafts outside of the nucleus, suggesting the existence of a different role for DNA-PKcs in cytoplasmic cellular signaling (Lucero et al., 2003). In support of these diverse findings, we demonstrate that DNA-PKcs interacts with mEAK-7 to regulate mTOR signaling, predominantly through S6K2.

In mini pigs, mTOR signaling is enhanced in salivary glands 5 days after treatment with X-ray irradiation (Zhu et al., 2016). In addition, PI3K and mTOR are essential regulators of radiation resistance in prostate cancer cells (Chang et al., 2014) as dual PI3K-mTOR inhibitors re-sensitize cancer cells to radiation treatment (Mukherjee et al., 2012). Here, we demonstrate that mEAK-7 is required for the sustained activity of mTOR signaling following X-ray irradiation damage. Continued investigation of mEAK-7 and other molecular machinery that regulates IR damage-mediated activation of mTOR signaling will allow for the creation of targeted inhibitors promoting radiation re-sensitization.

As the role of mEAK-7 is further studied in the context of human disease, its unique role in nutrient-sensing and DNA damage response (Figure 6F) will likely expand. S6K2 is a crucial component of mTOR signaling that has been overlooked (Pardo and Seckl, 2013). Yet, many studies demonstrate that S6K2 is associated with human diseases, including non-small-cell lung cancer (Pardo et al., 2006) and late-stage breast cancer (Karlsson et al., 2015). We determined that there are high mEAK-7 protein levels in the tumors and lymph nodes of patients with metastatic cancer, that mEAK-7^high^ patients have poor prognoses, and that mEAK-7 is essential for self-renewal and radioresistance. To determine the evolutionary benefit that cancer cells gain from upregulating mEAK-7, future research should be focused on elucidating the mechanisms allowing tumorigenesis in a broader range of cancers. Likewise, development of mEAK-7 inhibitors may benefit patients with metastatic cancers that demonstrate aberrant mTOR signaling associated with high levels of mEAK-7.

### Limitations of the Study

Here, we report that DNA-PKcs is a binding partner of the mEAK-7-mTOR complex. Some pitfalls of these studies are that we lack animal models that could recapitulate human disease. As most of our work is to identify novel binding partners and the detailed mechanism by which they interact, future studies will be required to examine the role of mEAK-7 *in vivo.* Also, our inhibitor studies against DNA-PKcs, although severalfold more specific to DNA-PKcs versus mTOR, could yield some off-target effects and DNA-PKcs knockout cell lines may not recapitulate all of physiology, suggesting that genetic approaches *in vivo* are required to understand the role of DNA-PKcs binding to mTOR to form mTORC3. Thus the role of this alternative complex to canonical mTOR signaling requires further study in animal models where mTOR signaling is required for eukaryotic development and disease progression.

## Methods

All methods can be found in the accompanying Transparent Methods supplemental file.

## Supplemental Information

Document S1. Transparent Methods and Figures S1–S6

Table S1. Immunoprecipitation-Mass Spectrometric Analysis of HA-mEAK-7 Extended full list of proteins from IP-MS experiment for Figure 5A.

Table S2. Detailed Patient Information from US Biomax TMAs Detailed patient information regarding TMAs analyzed for Figures 2A–2D and S2.
