# Supplementary material for: mEAK-7 Forms an Alternative mTOR Complex with DNA-PKcs in Human Cancer
Source: iScience. 2019 Jun 25;17:190–207. doi: 10.1016/j.isci.2019.06.029 (PMC6614755; doi:10.1016/j.isci.2019.06.029)
Supplement: Document S1. Transparent Methods and Figures S1–S6 [file mmc1.pdf]

ISCI, Volume *17*

Supplemental Information

mEAK-7 Forms an Alternative mTOR Complex with DNA-PKcs in Human Cancer

Joe Truong Nguyen, Fatima Sarah Haidar, Alexandra Lucienne Fox, Connor Ray, Daniela Baccelli Mendonça, Jin Koo Kim, and Paul H. Krebsbach

SUPPLEMENTAL INFORMATION

- Transparent Methods
- Figure S1, related to Figure 1: *MEAK7* expression patterns in normal human tissues and cancer patients.
- Figure S2, related to Figure 2 and Figure 3: Normal lymph tissue analysis, H1975 cancer stem cell analysis, and differential cell density clongenicity assay.
- Figure S3, related to Figure 3 and Figure 4: Colony formation assay with second mEAK-7 siRNA, spheroid formation assay with differential cell density or second mEAK-7 siRNA, and mEAK-7 effect of Noxa expression by X-ray irradiation in H1299 cells.
- Figure S4, related to Figure 5 and Figure 6: DNA damage mediated S6K1/2 signaling, and DNA-PKcs binding to S6K1, and second mEAK-7 siRNA effect on DNA damage or nutrient induced S6K2 activation
- Figure S5, related to Figure 6: MDA-MB-231 cell data on mEAK-7 and X-ray irradiation-mediated mTOR signaling.
- Figure S6, related to Figure 6: Dose-dependent analysis of NU7441 on IR-mediated mTOR signaling and DNA-PKcs, mTOR, and PI3K inhibitor study after X-ray irradiation.
- Table S1, related to Figure 5: Immunuoprecipitation-mass spectrometry analysis of mEAK-7.
- Table S2, related to Figure 2: Detailed patient information from US Biomax Tissue Microarrays.

TRANSPARENT METHODS

Cell lines

H1299 and H1975 are non-small cell lung carcinoma cell lines obtained from ATCC. MDA-MB-231 is a triple negative breast carcinoma cell line obtained from ATCC. DNA-PKcs^{WT} and DNA-PKcs$^{-/-}$ HEK-293T cells were kindly gifted from Dr. Kathryn Meek, a Professor at Michigan State University. H1299 cells (ATCC® CRL-5803™) were derived from a 43 year old male, Caucasian. H1975 cells (ATCC® CRL-5908™) were derived from an unknown aged, female. MDA-MB-231 (ATCC® HTB-26™) cells were derived from a 51 year old female Caucasian. HEK-293T cells (ATCC® ACS-4500™) were derived from an unknown aged, human embryonic kidney tissue. All cell lines are of human origin.

Statement of regulatory oversight and approval

The University of Michigan Institutional Review Boards is the committee approving the cell line experiments, approving the use of human samples from US Biomax, and confirming that all experiments conform to the relevant regulatory standards. It is not clear to what extent that sex, gender, and age affected data presented in human tissue microarrays obtained from US Biomax. The influence or association between these characteristics would require further assessment, with regards to the conclusions of this manuscript. Detailed information regarding patient samples from US Biomax can be accessed in Table S2.

Cell culture

Cell culture: Cell lines were grown in Dulbecco's minimal essential medium (DMEM, Thermo Fisher Scientific (TFS): cat# 11995-073), without antibiotics/antimycotics and supplemented with a concentration of 10% fetal bovine serum (FBS, TFS: cat# 10437-036, Lot # 1399413) at 37°C in 5.0% CO_2 incubator. Cells were grown in Falcon™ Tissue Culture Treated Flasks T-75 (Fisher Scientific (FS): cat# 13-680-65) until 75% confluent and split with Trypsin-EDTA 0.25% (TFS: cat# 25200-056) for 5 min in the 37°C cell incubator. Cells were washed 1x with PBS and resuspended in 10% FBS containing DMEM. Cells were counted with the LUNA™ Automated Cell Counter (Logos Biosystems (LB): cat# L10001) utilizing LUNA™ Cell Counting Slides (LB: cat# L12003) and AO-PI dye (LB: cat# F23001).

Small interfering RNA or plasmid transfection

Cells were seeded at a density of 500,000 cells per 60 mm TCP and grown for 24 hours. For siRNA transfection, Lipofectamine® RNAiMAX Transfection Reagent (TFS: cat# 13778-150) was incubated with Opti-MEM® I Reduced Serum Medium (TFS: cat# 31985-070) and 100 nM siRNA was incorporated before introduction to cells at 100 nM concentration. For plasmid transfection, FuGENE® 6 Transfection Reagent (Promega: cat# E2691) was incubated with Opti-MEM I Reduced Serum Medium and 2 µg plasmids were incorporated before introduction into cells. For dual transfection, we added both solutions. siRNAs used were as follows: mEAK-7 #1 siRNA (TFS: ID# s33640). mEAK-7 #2 siRNA (TFS: ID# HSS126697). S6K1 #1 siRNA (TFS: ID#

s12282). S6K1 #2 siRNA (TFS: ID# s12283). S6K2 #1 siRNA (TFS: ID # s12287). S6K2 #2 siRNA (TFS: ID # s12286). Control siRNA (TFS: cat# 4390843). Plasmids were purchased from Addgene. HA-S6K2 plasmid: pcDNA3-S6K2-WT was a gift from John Blenis (Addgene plasmid # 17729).

X-ray irradiation protocol

After appropriate treatment of cells with siRNA, plasmids, chemical compounds, nutrients, etc, we subjected the cells to X-ray irradiation. The machine is a Polaris IC-320 SC-500 series 2 from Kimtron. The dose rate is set at 4.5223Gy/min. X-ray irradiation of cells was done via the 1.5mm Aluminum filter, with a cone which has a 20x20cm beam pattern and a 50 cm FSD (radiation distance). X-ray irradiation applied is either 10 gy or 20 gy.

Immunofluorescence

Deparaffinization and rehydration steps were as follows: xylene for 10 minutes, 100% ethanol for 5 minutes, 95% ethanol for 5 minutes, 70% ethanol for 5 minutes, Milli Q water for 10 minutes, and 1x PBST for 10 minutes. Antigen retrieval steps were as follows: slides were placed in the slide holder to pressure cooker immersed in 10 mM citric acid (pH 6.0). Then, slides were placed in the microwave and cooked at full power for 12.5 minutes, finishing when pressure valve has been up for 1 min. Pressurized steam was exhausted from the pressure cooker. The pressure cooker and slides were cooled under running water for 15 minutes. Slides were washed with 1x PBS for 10

minutes. Slides were permeabilized for 10 minutes with 1x PBS with 0.4%Triton-X. Slides were blocked with 2.5% bovine serum albumin and 1% Tween20 in 1x TBS. Slides were incubated overnight at 4˚C with primary antibody. Next, slides were washed with PBS and incubated in secondary antibodies for 1 hour at room temperature. Slides were washed with PBS with DAPI for 10 minutes. Prolong Gold Antifade with DAPI was used to mount slides (Fisher cat# P36935). Nikon Ti Eclipse Confocal Microscope (60x with oil magnification) was used to capture images. Images were captured with or without 3x digital zoom, 1/32 frames per second, 1024x1024 image capture, 1.2 Airy Units, 2x line averaging, appropriate voltage and power settings optimized per antibody. No image modification was performed, except image sizing reduction for figure preparation. Quantitative analyses were completed via Nikon Analysis Software, with the data analysis and images representing the average of 3 fields of view and more than 50% of the tissue core. NSCLC tissue microarray used for protein level detection of mEAK-7 and p-S6 was purchased from US Biomax (cat# HLug-Squ090Lym-01). Healthy lymph tissue microarray used for protein level detection of mEAK-7 and p-S6 was purchased from US Biomax (cat# LN802A). NSCLC tissue microarray used for patient survival was purchased from US Biomax (cat# HLug-Squ150Sur-02). Primary antibodies for immunofluorescence were as follows: mEAK-7 (Santa Cruz Biotechnology (SCB) cat# sc-247321) and ($Ser^{240/244}$) p-S6 ribosomal protein (D68F8) XP® (Cell Signaling Technologies (CST): cat#5364S). All antibodies were used at 1:1,000 with a working volume of 1.5 mL in 5% BSA in PBS, unless noted otherwise. Secondary antibodies for immunofluorescence were as follows: Donkey anti-Goat IgG

Alexa Fluor® 647 (TFS: cat# A-21447), Anti-rabbit IgG (H+L), and F(ab')2 Fragment Alexa Fluor® 488 Conjugate (CST: cat# 4412S). All antibodies were used at a concentration of 1:1,000, with a working volume of 1.5 mL in 5% BSA in PBS. DAPI stain was used for DNA staining.

Immunoblot analysis

Cells were lysed in cold NP40 lysis buffer (50 mM Tris, 150 mM NaCl, and 1.0% NP-40 at pH 8.0). 50 µg of protein lysate was separated with Novex® Tris-Glycine SDS Running Buffer 10X (TFS: cat# LC2675-4) and Novex™ WedgeWell™ 4-20% Tris-Glycine Gels (TFS; cat# XP04205BOX), NuPAGE™ 3-8% Tris-Acetate Protein Gels (TFS; cat# EA03785BOX). Proteins were transferred to PVDF membranes. 4-20% gels were used for proteins 100 kDa and below, while 3-8% gels were used for proteins 100 kDa and above. Primary antibodies were incubated with membranes overnight at 4°C, and secondary antibodies were incubated with membranes at room temperature for 1 hour. Membranes were incubated with SuperSignal™ West Pico Chemiluminescent Substrate (TFS; cat# 34078) or Femto (TFS; cat# 34095) for film capture on HyBlot CL autoradiography film (Denville Scientific: cat# e3018). Primary antibodies were as follows: α-mEAK-7 (KIAA1609) mouse monoclonal antibody clone OTI12B1 (formerly 12B1) was obtained from Origene Technologies (OT; cat# TA501037, lot A01). All antibodies from Cell Signaling Technologies (CST) are rabbit: α-glyceraldehyde-3-phosphate dehydrogenase (CST: cat# 2118S), α-tubulin (CST: cat# 2144S), α-phospho-S6 ribosomal protein ($Ser^{240/244}$) (CST: cat# 2215S), α-phospho-S6 ribosomal

protein (Ser$^{235/236}$) (CST: cat# 2211S), α-S6 ribosomal protein (CST: cat# 2217S), α-phospho-p70 S6 kinase (Thr^{389}) (CST: cat# 9234S), α-S6K1 (CST: cat# 2708S), α-S6K2 (CST: cat# 14130S), α-mTOR (CST: cat# 2983S), α-HA-tag mouse (CST: cat# 2367S), α-HA-tag rabbit (CST: cat# 3724S), α-(Ser^{65}) p-4E-BP1 (CST: cat# 9451S), α-(Thr$^{37/46}$) p-4E-BP1 (CST: cat# 9459S), α-(Thr^{70}) p-4E-BP1 (CST: cat# 13396S), α-4E-BP1 (CST: cat# 9452S), α-N-cadherin (CST: cat# 13116S), α-noxa (CST: cat# 14766S), α-Cleaved PARP (CST: 5625S), α-(Thr^{68}) p-Chk2 (CST: cat# 2197S), α-Chk2 (CST: cat# 3440S), α-(Ser^{2448}) p-mTOR (CST: cat# 2971S). Concentration of antibodies: p-S6, S6, and 4E-BP1 used at 1:3,000 dilution and remainder at 1:1,000 dilution in 5% BSA in 1X TBST buffer with 0.04% sodium azide. Secondary antibodies for immunoblot analysis: 1:4,000 dilution for α-mouse IgG (Promega; cat# W4021). 1:7,500 dilution for α-rabbit (Promega; cat# W4011), and 1:2,000 dilution for α-rabbit light chain specific antibody (Abcam: cat# ab99697) only for S6K2 IP experiments.

Chemical Inhibitors

All chemical were resuspended in DMSO, according to manufacturer recommendations. Rapamycin (CST; cat# 9904S), LY293002 (CST; cat# 9901S), NU7441 (Tocris Biotechne; cat# 3712). All inhibitors were applied to cells for at least 2 hours, unless stated otherwise in the manuscript.

Immunoprecipitation (IP) analysis and mass spectrometry

After siRNA and/or plasmid transfection, cells were harvested in 1% NP40 lysis buffer or CHAPS lysis buffer (FIVEphoton Biochemicals (FB): cat# CIB-1) supplemented with protease inhibitors (FB: cat# PI-1) and phosphatase inhibitors (FB: cat# PIC1). For antibody-bead conjugation, 1 to 2 µg of antibodies and 50 µL of mixed Protein A/G PLUS-Agarose (SCB; Cat # sc-2003) were incubated for 1 hour on vertical shaker at 4°C. Afterwards, the antibody-bead mix was washed 3 times with 1x PBS. Next, 250 µg of protein in CHAPS buffer were incubated with the antibody-bead mix for 1.5 hours on vertical shaker at 4°C. After incubation, the antibody-bead conjugates were washed 3 times with 1x PBS. Beads were washed 3 times with 1x PBS, and 3x loading buffer with SDS was added to the bead mix, boiled, spun down, and utilized for immunoblot analysis. Immunoprecipitation-mass spectrometry: Samples were processed by the University of Michigan Proteomics core for IP/MS analysis and protocols can be found on their webpage. Samples submitted to the core were pooled from 3x reactions of HA-mEAK-7 in H1299 cells, as described above. Full excel sheet supplied as Table S1. Antibodies used for immunoprecipitation reactions were as follows: Anti-HA epitope tag polyclonal goat IgG Antibody (Novus Biologicals: cat# NB600-362), polyclonal goat IgG antibody (SCB: cat# sc-2028), α-S6K2 (CST: cat# 14130S), α-mTOR (CST: cat# 2983S), mEAK-7 (SCB, cat# sc-247321).

Cell Invasion assay

After siRNA transfection and X-ray irradiation, cells were trypsinized and 50,000 cells were seeded onto Corning® Matrigel® Invasion Chamber 24-Well Plate 8.0 Micron

(Corning: cat# 354480), with 1 mL of DMEM^{-AAs} without FBS within the top chamber, and 1 mL of 10% FBS-containing DMEM medium on the bottom of the plate. After 24 hours, we processed the samples with Hema 3™Stat Pack (Fisher: cat# 123-869), according to manufacturer specifications. Images were captured with a stereoscope, attached to a digital camera. Brightness and contrast were adjusted, as needed. Analysis was conducted via student's t-test. % invasion was counted on 6 individual experiments per condition as: # cells adhered to the bottom chamber divided by # cells seeded total.

Comet Assay

Lysis Buffer, Alkaline Solution, and Eletrophoresis Running Solution were prepared according to manufacturer's instructions. Solutions were stored at 4°C. Oxiselect Comet Agarose (Cell Bio Labs (CBL): #235002) was heated to 95°C for 20 minutes, then placed in 37°C water bath until use. Cells were grown according to experimental procedures for siRNA treatment. Two days post siRNA treatment, cells were subjected to no treatment, 2 gy, and 6 gy X-ray irradiation. Cells were trypsinized and resuspended at a concentration of $1x10^5$ cells/mL in cold PBS. Then, 10 µL of cell suspension was mixed with 90 µL of comet agarose. After mixing thoroughly, 75 µL of this mixture was transferred to the OxiSelect Comet Slide (CBL: #STA-352). Slides were placed in the dark at 4°C for 15 minutes. Slides were transferred to a small basin containing pre-chilled Lysis buffer and placed at 4°C for 60 minutes in the dark. Lysis solution was aspirated from the basin, replaced with pre-chilled Alkaline solution, and

placed at 4°C in the dark for 30 minutes. Then, Alkaline solution was aspirated and replaced with pre-chilled TBE Electrophoresis solution. After 5 minutes, TBE Electrophoresis solution was aspirated and replaced with new TBE Electrophoresis solution. Slides were transferred to a horizontal electrophoresis chamber and the well was filled with enough TBE Electrophoresis solution to fully cover the slides. Voltage was applied for 45 minutes at 20 volts. After electrophoresis, slides were transferred to a small basin containing pre-chilled DI H2O, and the slides were fully immersed. After 2 minutes, the DI H2O was aspirated and replaced. This rinse was repeated twice. After the third rinse, slides were immersed in cold 70% ethanol for 5 minutes, then removed from the basin and allowed to air dry. Once the agarose was dried fully, 100 µL of diluted Vista Green DNA dye (CBL: cat# 235003, diluted 1:10,000 in TE buffer) was added to each well and allowed to incubate at room temperature for 15 minutes. Slides were imaged with Nikon Ti Eclipse Confocal Microscope at 10x magnification lens to capture images. Images were captured at 1/8 frames per second, 1024x1024 image capture, 1.2 Airy Units, 2x line averaging, appropriate voltage and power settings for FITC (488 nm). No image modification was performed, except image sizing reduction for figure preparation.

Cancer Stem Cell sorting

Cells were trypsinized and resuspended in DMEM medium with 10% FBS and counted on Luna cell counter (Logos Biosystems (LB): cat# L10001) using Acridine Orange/Propidium Iodide dye (LB: cat# F23001) for viability. $1x10^7$ cells were filtered

into each of 5 labeled 50 mL falcon tubes, through a 40 µm filter. $1x10^7$ cells were filtered into each of 5 labeled 5 mL round bottom tubes (Falcon: cat# 352235) to be used as single-color controls. Tubes were centrifuged at 1,000 rpm for 4 minutes at 4°C and supernatant was discarded. One 5 mL single color control tube was resuspended with 200 μL PBS with 10% FBS to be used as a negative control. Three of the other single-color control tubes were resuspended with 200 µL PBS with 10% FBS, and single color dyes were added as detailed: 2 µL DAPI (Thermo Fisher: cat# D1306), 25 µL CD90 (Biolegend: cat# 328107), and 10 µL CD44 (BD Biosciences: cat# 559942). The final single-color tube was used for Isotype control. This pellet was resuspended with 191 µL PBS with 10% FBS and 2 µL DAPI, 2 µL APC Isotype (BD Biosciences: cat# 340442), and 5 µL FITC Isotype (BD Bioscience: cat# 555909) were added. Cells in the 50 mL tubes were resuspended with 10 mL PBS with 10% FBS. The stain master mix was added to each tube to be sorted. Tubes were placed on a rack and incubated at 37°C for 30 minutes. Cells were rinsed with 1x PBS and resuspended 1x PBS containing 3% FBS. Place all tubes on ice until samples will be run on Flow Cytometer (Sony: Cat# SH800).

Clonogenicity Assay

Cells were grown according to experimental procedures for siRNA treatment. Two days post siRNA treatment, cells were subjected to no treatment, 2 gy, and 6 gy X-ray irradiation. 10,000 H1975 cells or 2,500 H1299 cells were plated into 60 mm dishes with 2 mL DMEM with 10% FBS. Cells were cultured in the incubator for 10 days (until

control colonies contained >50 cells), and new media and new siRNA were added every 4 days. Cells were fixed with 2 mL of fixation mix (glacial acetic acid (Sigma-Aldrich 537020) and methanol (Sigma-Aldrich 67-56-1), at a 1:7 ratio) for 2-3 minutes at room temperature and incubated with 2 mL Crystal Violet (Sigma-Aldrich C6158-50G, diluted to 0.5% in Milli-Q water) for 2 hours at room temperature. After 2 hours, the Crystal Violet was removed and the dishes were rinsed with 2 mL of media (no FBS added), pipetting vigorously to dislodge cells. Dishes were rinsed carefully in DI water and placed on paper towel to dry for 2-3 days. Once plates were dry, colonies were counted and recorded.

Spheroid Formation Assay

Cells were grown according to normal experimental procedures for siRNA treatment (n=6). Two days post siRNA treatment, cells were subjected to no treatment, 2 gy and 6 gy X-ray irradiation. 10,000 H1975 cells or 5,000 H1299 cells were plated into ultralow attachment plates (Costar: cat# 3471). 2 mL of Serum Free Medium (435 mL MEBM Medium (Lonza: cat# CC-3151), 10 mL B27 (Gibco: cat# 17504-044), 5 mL Pen/Strep (Gibco: cat# 15070), 5 mL Lipid Concentrate (Gibco: cat# 11905-031), 2.5 mL Insulin (Sigma-Aldrich: cat# I6634), 10 µg EGF (BD Biosciences: cat# 354052), 10 µg bFGF (BD Bioscience: cat# 354060), 500 µg Hydrocortisone (Sigma-Aldrich: cat# H4001), 500 µL 100 mM ß-mercaptoethanol (Sigma-Aldrich: cat# M3148), 2 mg Cholesterol (Sigma-Aldrich: cat# C4951)) were added to each well. Cells were grown for 2 weeks, adding 500 µL of Serum Free Medium every 4 days to ensure cells have adequate nutrients.

After 2 weeks, the number of spheres growing in each well (spheres must have defined, circular edges and be made of at least 10 cells) was counted, and representative images of spheres from each treatment group were taken.

Statistical analysis and reproducibility

GTEx data acquisition, processing, and statistical analysis information can be found on “gtexportal.org”. cBioportal was accessed by searching for “*MEAK7*” or “*MEAK7*: gain amp”. Patient derived data from Oncomine were analyzed via unpaired student’s t-test. Patient derived tissue microarray data were analyzed via paired Mann-Whitney’s U-test. 2D/3D clonogenicity assay, comet assay, cell proliferation, cell migration, and cell size were analyzed via paired student’s t-test. Immunoblot and immunoprecipitation assays were repeated at least thrice in all cell lines.

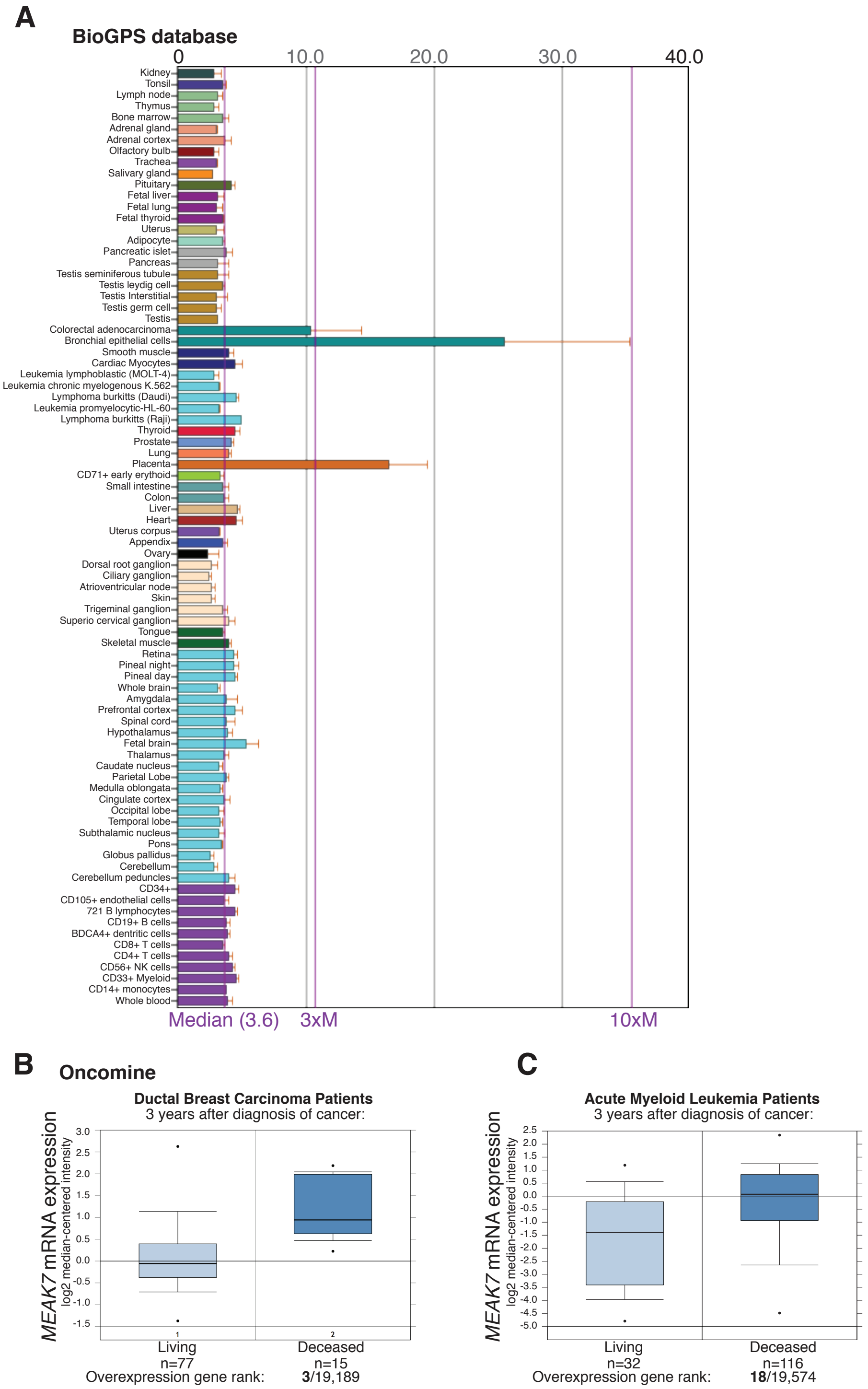
A
BioGPS database
0
10.0
20.0
30.0
40.0
Kidney
Tonsil
Lymph node
Thymus
Bone marrow
Adrenal gland
Adrenal cortex
Olfactory bulb
Trachea
Salivary gland
Pituitary
Fetal liver
Fetal lung
Fetal thyroid
Uterus
Adipocyte
Pancreatic islet
Pancreas
Testis seminiferous tubule
Testis leydig cell
Testis Interstitial
Testis germ cell
Testis
Colorectal adenocarcinoma
Bronchial epithelial cells
Smooth muscle
Cardiac Myocytes
Leukemia lymphoblastic (MOLT-4)
Leukemia chronic myelogenous K.562
Lymphoma burkitts (Daudi)
Leukemia promyelocytic-HL-60
Lymphoma burkitts (Raji)
Thyroid
Prostate
Lung
Placenta
CD71+ early erythoid
Small intestine
Colon
Liver
Heart
Uterus corpus
Appendix
Ovary
Dorsal root ganglion
Ciliary ganglion
Atrioventricular node
Skin
Trigeminal ganglion
Superio cervical ganglion
Tongue
Skeletal muscle
Retina
Pineal night
Pineal day
Whole brain
Amygdala
Prefrontal cortex
Spinal cord
Hypothalamus
Fetal brain
Thalamus
Caudate nucleus
Parietal Lobe
Medulla oblongata
Cingulate cortex
Occipital lobe
Temporal lobe
Subthalamic nucleus
Pons
Globus pallidus
Cerebellum
Cerebellum peduncles
CD34+
CD105+ endothelial cells
721 B lymphocytes
CD19+ B cells
BDCA4+ dentritic cells
CD8+ T cells
CD4+ T cells
CD56+ NK cells
CD33+ Myeloid
CD14+ monocytes
Whole blood
Median (3.6)
3xM
10xM
B
Oncomine
Ductal Breast Carcinoma Patients
3 years after diagnosis of cancer:
MEAK7 mRNA expression
log2 median-centered intensity
Living
n=77
Deceased
n=15
Overexpression gene rank: 3/19,189
C
Acute Myeloid Leukemia Patients
3 years after diagnosis of cancer:
MEAK7 mRNA expression
log2 median-centered intensity
Living
n=32
Deceased
n=116
Overexpression gene rank: 18/19,574

Figure S1, related to Figure 1. *MEAK7* is expressed at basal levels in many normal human tissues, but significantly overexpressed in human cancer patients with mortality. (**A**) BioGPS analysis of *MEAK7* in human tissues and cells. (**B, C**) Oncomine analysis of *MEAK7* gene expression of patients with (b) ductal breast carcinoma (P=2.72x10^{-6}, Fold Change: 2.136) and (c) acute myeloid leukemia (P=7.99x10^{-6}, Fold Change: 2.655).

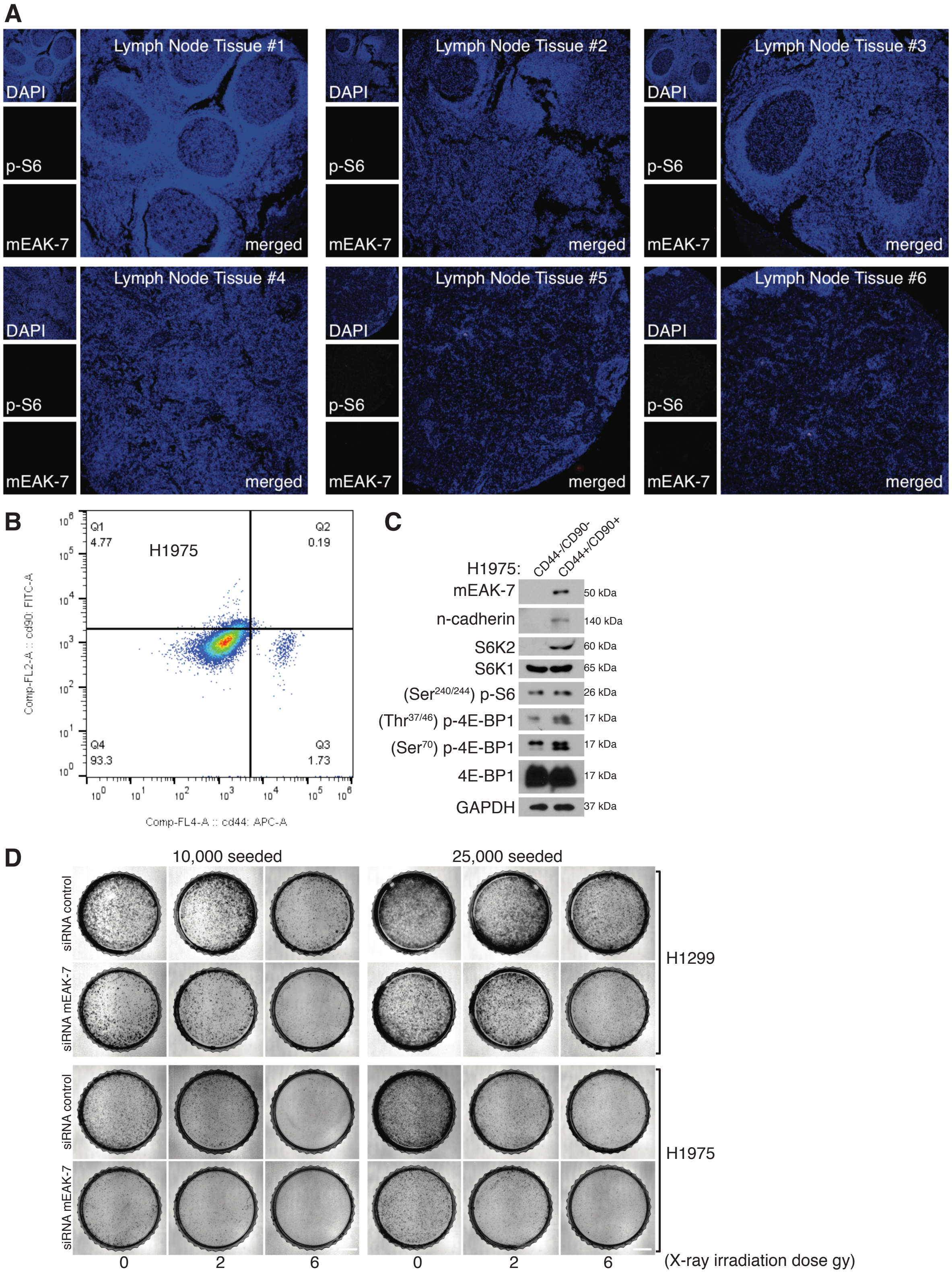
A
Lymph Node Tissue #1
Lymph Node Tissue #2
Lymph Node Tissue #3
Lymph Node Tissue #4
Lymph Node Tissue #5
Lymph Node Tissue #6
DAPI
p-S6
mEAK-7
merged
B
H1975
Q1 4.77
Q2 0.19
Q3 1.73
Q4 93.3
Comp-FL2-A :: cd90: FITC-A
Comp-FL4-A :: cd44: APC-A
C
H1975:
CD44-/CD90-
CD44+/CD90+
mEAK-7 50 kDa
n-cadherin 140 kDa
S6K2 60 kDa
S6K1 65 kDa
(Ser240/244) p-S6 26 kDa
(Thr37/46) p-4E-BP1 17 kDa
(Ser70) p-4E-BP1 17 kDa
4E-BP1 17 kDa
GAPDH 37 kDa
D
10,000 seeded
25,000 seeded
siRNA control
siRNA mEAK-7
H1299
H1975
0
2
6
(X-ray irradiation dose gy)

Figure S2, related to Figure 2 and Figure 3. Normal lymph tissue analysis, H1975 cancer stem cell analysis, and differential cell density clongenicity assay. (**A**) 6 representative sections of US Biomax tissue microarray LN802a was analyzed using the antibodies against mEAK-7 and p-S6. (**B**) Flow sort diagram depicting the CD44+/CD90+ cell population in H1975 cells. (**C**) Immunoblot analysis of CD44-/CD90- and CD44+/CD90+ H1975 cells for mEAK-7 and mTOR signaling. (**D**) H1299 and H1975 cells were treated with control or mEAK-7 siRNA, X-irradiated at 2 or 6 gy, and 10,000 or 25,000 cells were seeded into 60 mm TCPs and grown for 10 days. White bars denote 2.5 mm. This experiment was repeated at least 6 times.

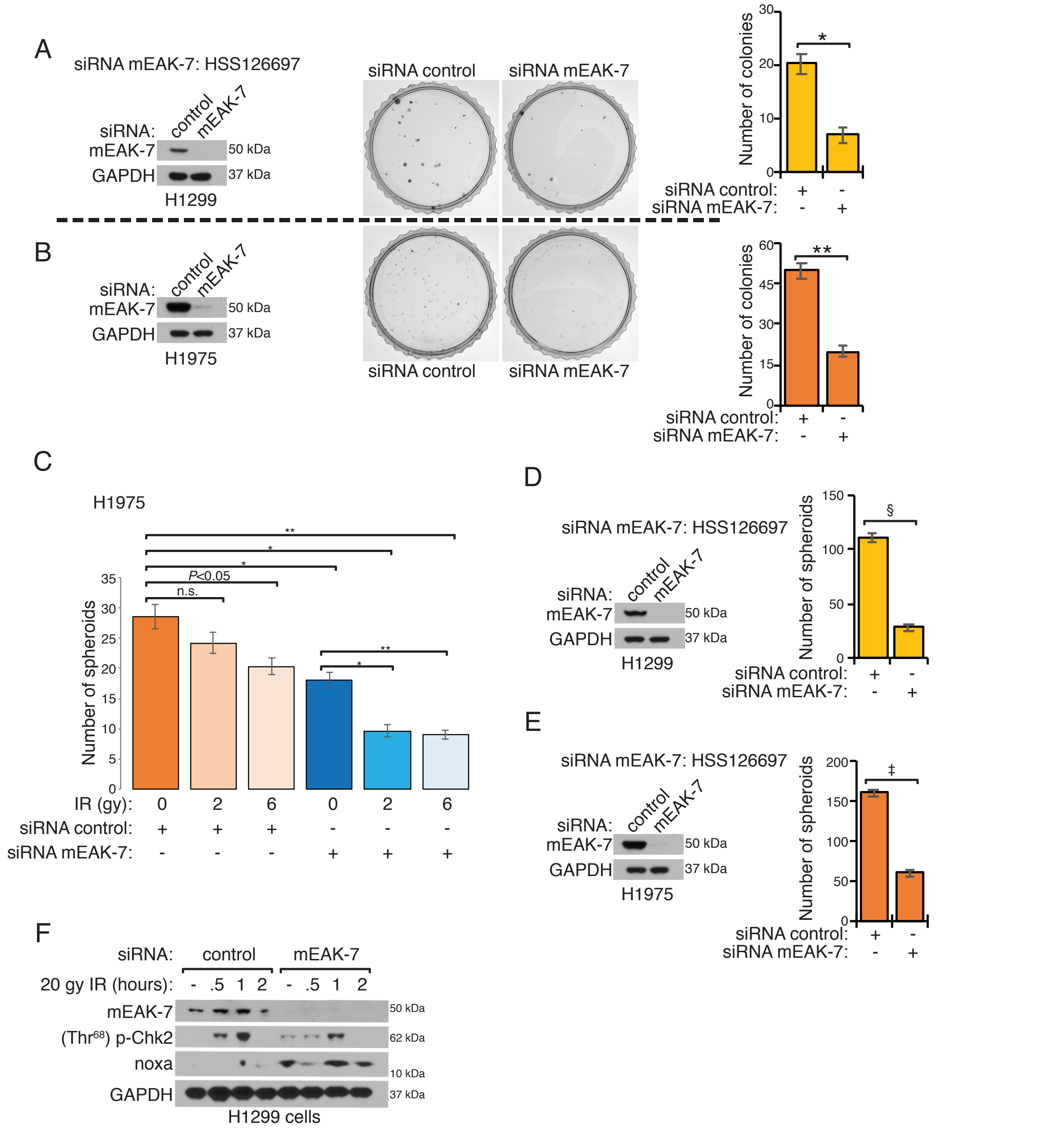

Figure S3, related to Figure 3 and Figure 4. Colony formation assay with second mEAK-7 siRNA, spheroid formation assay with differential cell density or second mEAK-7 siRNA, and mEAK-7 effect of Noxa expression by X-ray irradiation in H1299 cells. (**A**) H1299 cells were treated with control or mEAK-7 siRNA (ID: HSS126697) for 48 hours, and 500 cells were seeded into 60 mm TCPs and grown for

10 days. Quantification of colony formation and analysis via student's t-test (n=6) of (A), **P*<0.01. (**B**) H1975 cells were treated with control or mEAK-7 siRNA (ID: HSS126697) for 48 hours, and 500 cells were seeded into 60 mm TCPs and grown for 10 days. Quantification of colony formation and analysis via student's t-test (n=6) of (B), **P*<0.01, ***P*<0.001. (**C**) H1975 cells were treated with control or mEAK-7 siRNA, subjectd to no treatment, 2 gy, or 6 gy of X-ray irradiation, and 5,000 cells were seeded into 60 mm ultra-low attachment plates and grown for 10 days. Quantification of spheroid formation and analysis via student's t-test (n=6), **P*<0.01, ***P*<0.001. (**D**) H1299 cells were treated with control or mEAK-7 siRNA (ID: HSS126697), and 2,500 cells were seeded into 60 mm ultra-low attachment plates and grown for 10 days. Quantification of spheroid formation and analysis via student's t-test (n=6) of (D), **P*<0.01, ***P*<0.001, ****P*<0.0001, ‡*P*<0.00001, §*P*<0.000001. (**E**) H1975 cells were treated with control or mEAK-7 siRNA (ID: HSS126697), and 5,000 cells were seeded into 60 mm ultra-low attachment plates and grown for 10 days. Quantification of spheroid formation and analysis via student's t-test (n=6) of (E), **P*<0.01, ***P*<0.001, ****P*<0.0001, ‡*P*<0.00001, §*P*<0.000001. (F) H1299 cells were treated with control or mEAK-7 siRNA and X-ray irradiated at 20 gy for 30 minutes, 1 hour, or 2 hours and analyzed for Noxa expression by DNA damage.

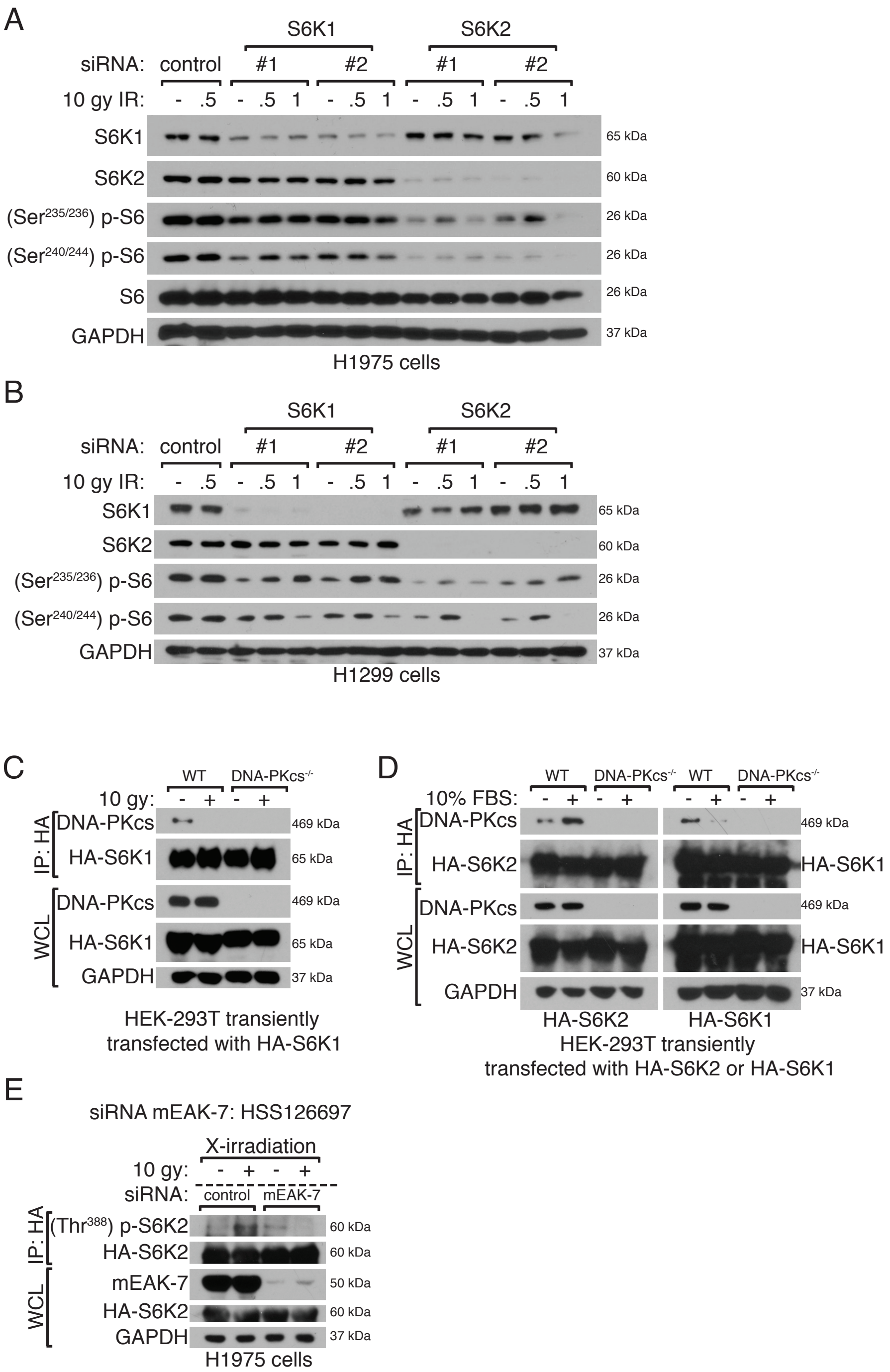

Figure S4, related to Figure 5 and Figure 6. DNA damage mediated S6K1/2 signaling, and DNA-PKcs binding to S6K1, and second mEAK-7 siRNA effect on DNA damage or nutrient induced S6K2 activation. (**A**) H1975 cells were treated with control, 2 unique S6K1, or 2 unique S6K2 siRNAs, then subjected to no treatment, 10

gy of X-ray irradiation for 30 minutes or 1 hour. Immunoblot analysis on mTOR signaling. (**B**) H1299 cells were treated with control, 2 unique S6K1, or 2 unique S6K2 siRNAs, then subjected to no treatment, 10 gy of X-ray irradiation for 30 minutes or 1 hour. Immunoblot analysis on mTOR signaling. (**C**) HEK-293T DNA-PKcs$^{+/+}$ or HEK-293T DNA-PKcs$^{-/-}$ cells were transiently transfected with or without pRK7-HA-S6K1-WT, then untreated or treated with 10 gy of X-ray irradiation for 1 hour. HA-S6K1 was immunoprecipitated to check DNA-PKcs interaction. This experiment was completed at least 3 times. (**D**) HEK-293T DNA-PKcs$^{+/+}$ or HEK-293T DNA-PKcs$^{-/-}$ cells were transiently transfected with pcDNA3-HA-S6K2 or pRK7-HA-S6K1-WT for 48 hurs. Next, cells were starved in DMEM^{-AAs} for 1 hour and reintroduced with 10% FBS in DMEM^{AAs} for 30 minutes. Cells were collected in CHAPS lysis buffer and immunoprecipitated with HA antibody. (**E**) H1975 cells were transiently transfected with pcDNA3-HA-S6K2 and control or mEAK-7 siRNA (ID: HSS126697) for 48 hours. Next, cells were untreated or treated with 10 gy of X-ray irradiation for 30 minutes, followed by IP of HA-S6K2, and probed for activated S6K2 signaling.

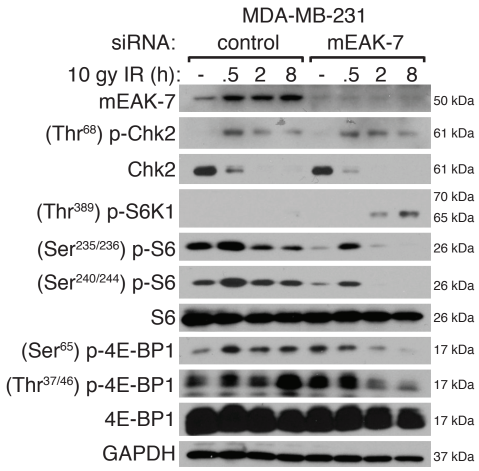

Figure S5, related to Figure 6. mEAK-7 is required for X-ray irradiation-mediated mTOR signaling in MDA-MB-231 cells. MDA-MB-231 cells were treated with control or mEAK-7 siRNA for 48 hours, X-irradiated at 10 gy for 30 minutes, 2 hours, and 8 hours and processed for mTOR signaling. This experiment was completed at least 3 times.

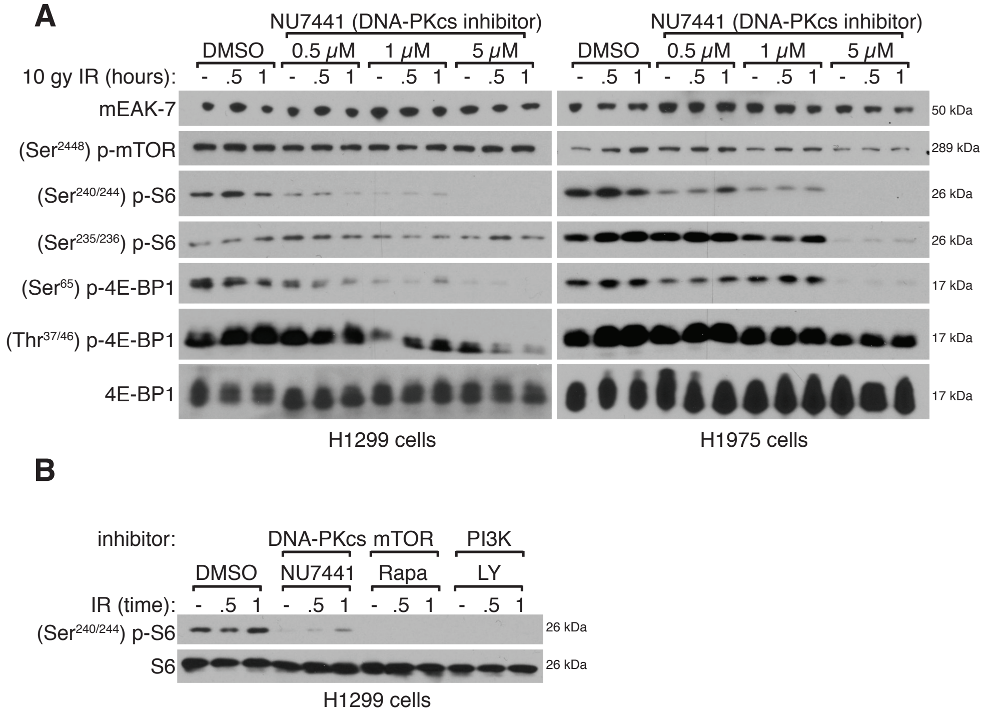

Figure S6, related to Figure 6. Dose-dependent analysis of NU7441 on IR-mediated mTOR signaling and inhibition of DNA-PKcs, mTOR, and PI3K significantly decreased IR-mediated mTOR signaling in H1299 cells. (**A**) H1299 and H1975 cells were treated with DMSO or 0.5 μM, 1 μM, 5 μM NU7441 for 2 hours before treated with X-ray irradiation at 10 gy for 30 minutes and 1 hour. Immunoblot analysis on mTOR signaling. All experiments were repeated at least 3 times. 4E-BP1 was utilized as a loading control. (**B**) H1299 cells were treated with inhibitors of DNA-PKcs (5 μM NU7441, IC50 = 14 nM), mTOR (100 nM rapamycin, IC50 = 1 nM), and PI3K (50 μM LY249002, IC50 = 2.3 μM) for 1 hour before treated with X-ray irradiation at 10 gy for 30 minutes and 1 hour. Immunoblot analysis on mTOR signaling. We observed that inhibition of DNA-PKCS, mTOR, or PI3K significantly decreased IR-

mediated mTOR signaling. All experiments were repeated at least 3 times. S6 was utilized as a loading control.

Table S1. Immunoprecipitation-mass spectrometry analysis of HA-mEAK-7.

Extended full list of proteins from IP-MS experiment for Figure 5A.

Table S2: Detailed patient information from US Biomax TMAs. Detailed patient information regarding TMAs analyzed for Figure 2A-C, Figure 2D, and Figure S2.
